# USP35 regulates mitotic progression by modulating the stability of Aurora B

**DOI:** 10.1038/s41467-018-03107-0

**Published:** 2018-02-15

**Authors:** Jinyoung Park, Mi-Sun Kwon, Eunice EunKyeong Kim, Hyunsook Lee, Eun Joo Song

**Affiliations:** 1 Molecular Recognition Research Center, Korea Institute of Science and Technology, Hwarangno 14-gil 5, Seongbuk-gu, Seoul 02792 Korea; 2 Department of Biological Sciences & Institute of Molecular Biology and Genetics (IMBG), Seoul National University, Gwanak-Ro 1, Gwanak-Gu, Seoul 08826 Korea; 3 Biomedical Research Institute, Korea Institute of Science and Technology, Hwarangno 14-gil 5, Seongbuk-gu, Seoul 02792 Korea; 4 Division of Bio-Medical Science & Technology, KIST School, Korea University of Science and Technology, Daejeon, Seoul 02792 Korea

## Abstract

Although approximately 100 deubiquitinating enzymes (DUBs) are encoded in the human genome, very little is known about the DUBs that function in mitosis. Here, we demonstrate that DUB USP35 functions as a mitotic regulator by controlling the protein levels and downstream signaling of Aurora B and the depletion of USP35 eventually leads to several mitotic defects including cytokinesis failures. USP35 binds to and deubiquitinates Aurora B, and inhibits the APC^CDH1^-mediated proteasomal degradation of Aurora B, thus maintaining its steady-state levels during mitosis. In addition, the loss of USP35 decreases the phosphorylation of histone H3-Ser10, an Aurora B substrate. Finally, the transcription factor FoxM1 promotes the expression of USP35, as well as that of Aurora B, during the cell cycle. Our findings suggest that USP35 regulates the stability and function of Aurora B by blocking APC^CDH1^-induced proteasomal degradation, thereby controlling mitotic progression.

## Abstract (web summary)

Aurora B kinase is a member of the chromosomal passenger complex, which is an indispensable regulator of mitosis. Here the authors show that the deubiquitinating enzyme USP35 has a role in mitotic progression by inhibiting proteasomal degradation of Aurora B kinase, leading to its activation.

## Introduction

Deubiquitinating enzymes (DUBs) are proteases that cleave a single ubiquitin or polyubiquitin chains from target proteins. DUBs can affect protein–protein interactions and the localization or activity of a protein. DUBs display specificity towards particular chain types, e.g., lysine 11 (K11)- or lysine 48 (K48)-linked chains trigger the proteasomal degradation of target proteins while lysine 63 (K63) linkages typically facilitate the protein–protein interactions that are required for cell signaling although recent studies show increasing complexity of ubiquitin chains^1^. These activities have effects on cellular processes such as signal transduction, DNA damage repair, and cell cycle progression^2^. Some DUBs regulate mitotic progression via the deubiquitination of target substrates. For example, USP44 directly deubiquitinates CDC20 and counteracts the APC-driven disassembly of the Mad2–CDC20 complex, which regulates the proper mitotic timing and the spindle checkpoint function^3^. In addition, USP44 localizes to the centrosome and interacts with the centriole protein Centrin. The USP44–Centrin complex is required for proper centrosome separation, and the loss of this function results in aneuploidy^4^. Recently, USP33 has been reported to regulate centrosome biogenesis by deubiquitinating the centriole protein CP110^5^.

For successful mitosis, chromosomes, spindle microtubules, and membranes should move accurately to the proper site at the proper time^6^. These phenomena are mainly controlled by the chromosomal passenger complex (CPC), which regulates the entire process of mitosis including chromosome condensation, chromosome segregation, and cytokinesis. This complex is composed of the enzymatic component Aurora B kinase and the three regulatory and targeting components INCENP, survivin, and borealin^7^. Post-translational modifications, specifically the ubiquitination-induced alteration of the localization or degradation of Aurora B are critical to controlling its functions as a CPC protein kinase^8,9^. Degradation of Aurora B is achieved through the activation of APC/C E3 ligase. At the mitotic exit, the dephosphorylation of CDH1 and the degradation of the CDH1-binding protein MAD2L2 allow CDH1 to bind to APC/C, consequently ubiquitinating and degrading Aurora B^10^. However, how deubiquitination regulates Aurora B function has not been elucidated. Aurora B is regulated at mRNA levels by the FoxM1 transcription factor. Once FoxM1 binds to the promoter region of Aurora B in the late G2 phase, Aurora B expression is increased, thus synthesizing Aurora B protein rapidly and playing a role in mitosis^11^.

Here, we provide evidence regarding the crucial role of USP35 in mitosis control. We find that USP35 knockdown induces several mitotic defects and mitotic delay compared with controls. USP35 binds to and deubiquitinates Aurora B, which serves to stabilize and activate Aurora B. In addition, this reaction antagonizes the APC^CDH1^-dependent K11-linked ubiquitination of Aurora B. Finally, we determine that USP35 expression is regulated by FoxM1 as well as Aurora B in the cell cycle. Taken together, these data suggest that USP35 plays a critical role in the maintenance of the steady-state levels of Aurora B via blocking APC^CDH1^-induced ubiquitination, therefore, ensuring faithful mitotic progression.

## Results

### USP35 functions in mitosis

We previously analyzed the roles of DUBs in cell cycle control using siRNAs targeting approximately 70 human DUBs, and our study revealed several DUBs whose depletion resulted in either pre-mitotic arrest or spindle checkpoint bypass in Taxol-treated HeLa cells^12^. Out of these DUBs, we focused our investigation on the role of USP35 in this study. Recently USP35 is reported to localize to healthy polarized mitochondria and regulates the stability of MFN2 during PARK2-mediated mitophagy, thus participating in mitochondrial quality control^13^. In addition, USP35 functions as a tumor suppressor. When activated by miR let-7a, USP35 inhibits NF-κB activation by deubiquitinating and stabilizing ABIN-2, which subsequently inhibits tumor growth^14^. However, the functions of USP35 and the mechanisms regulating USP35 during mitosis remain largely unknown.

**Fig. 1:**
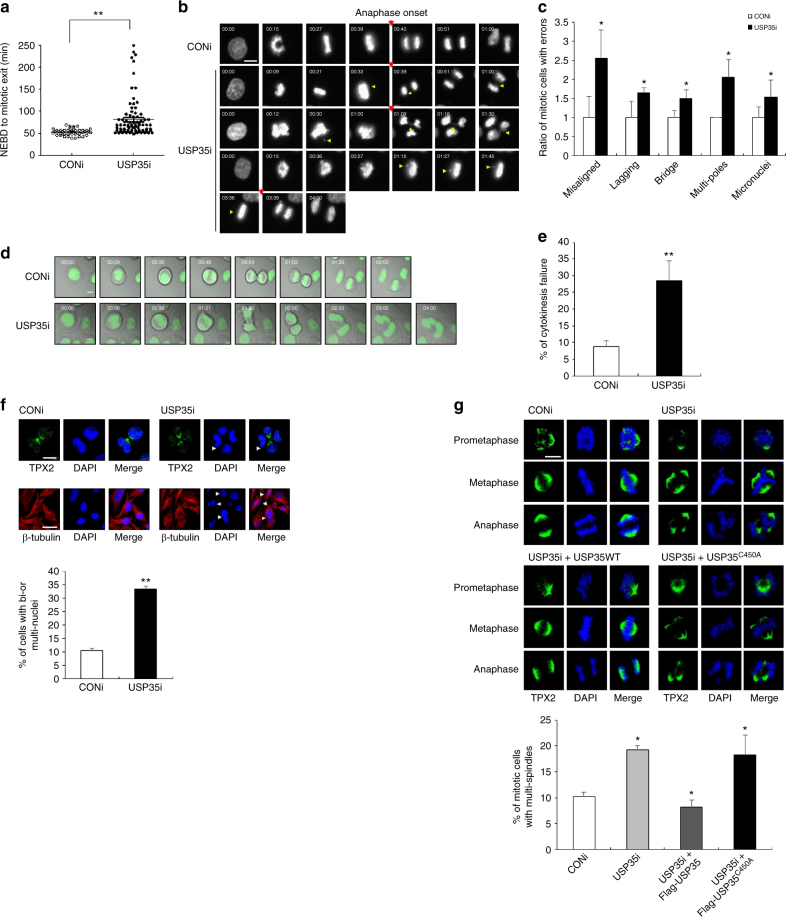
USP35 is required for faithful mitotic progression. **a**–**e** GFP-H2B-expressing HeLa cells transfected with a siRNA targeting USP35 (USP35i, *n* = 105) or a control siRNA (CONi, *n* = 64) were synchronized with thymidine and then released in a fresh medium. These cells were observed using time-lapse microscopy for 12 h, and images were captured every 3 min. The results were from three independent experiments. **a** The mitotic timing, defined as the time from NEBD to the mitotic exit, in the transfected cells. **b** Time-lapse microscopy of cells undergoing mitosis. Representative images are shown at the indicated times from NEBD (NEBD occurred at 00:00). The timing of anaphase onset is denoted by the red arrow. Yellow arrows indicate misaligned or lagging chromosomes, chromatin bridges, or improperly separated chromosomes. Scale bar = 10 μm. **c** Quantification of mitotic defects from time-lapse videos. The data are shown as ratios. **d** Time-lapse microscopy of cells undergoing mitosis and cytokinesis. Representative images were formed by merging phase contrast images and H2B-GFP fluorescence images. Scale bar = 10 μm **e** Quantification of cytokinesis failure from time-lapse videos. **f** HeLa cells were transfected with CONi or USP35i. Immunofluorescence staining was performed using a TPX2 antibody or a β-tubulin Cy3 antibody (top). Cells in bi- or multi-nuclei conditions were counted after immunofluorescence staining using a β-tubulin Cy3 antibody (bottom). One-hundred cells per group were examined from three independent experiments. Scale bar = 10 μm (top) or 20 μm (bottom). **g** HeLa cells were transfected with USP35i alone or in combination with Flag-USP35 or Flag-USP35^C450A^. Immunofluorescence staining was performed using a TPX2 antibody (top). Mitotic cells showing multi-spindles were counted after immunofluorescence staining (bottom). One-hundred cells per group were examined from three independent experiments. Scale bar = 10 μm. The data in parts **a**, **c**, **e**, **f**, and **g** represent the mean ± SD (*P < 0.05; **P < 0.005, *t*-test)

First, to confirm previous screening experiments using siRNAs, we transfected HeLa cells with siRNA targeting USP35 or control siRNA, and then treated them with Taxol. A microscopic analysis showed that Taxol-induced mitotic arrest occurred in the control cells, whereas USP35-deficient cells mostly displayed multi-lobed nuclei and severe nuclear abnormalities (Supplementary Fig. 1a). These morphologies illustrate that USP35 knockdown interferes with the spindle checkpoint function in Taxol-treated cells. To validate the effect of USP35 in mitosis further, we optimized live-cell imaging of USP35 siRNA or control siRNA-transfected HeLa cells expressing GFP-H2B. The USP35 level was decreased by USP35 siRNA while toxicity by siRNA transfection in the cells was not observed (Supplementary Fig. 1b). Monitoring the mitotic timing, defined as the time from nuclear envelope breakdown (NEBD) to the mitotic exit, in living cells using time-lapse microscopy revealed that the mitotic timing was delayed by USP35 knockdown (USP35i; 80.97 ± 4.784 min, *n* = 49) compared to the control siRNA treatment (CONi; 52.78 ± 1.024 min, *n* = 87) (these results obtained from three independent experiments are shown as mean ± SD) (Fig. 1a, Supplementary Movie 1 and 2). In the live-cell analyses, USP35-depleted cells showed a high-frequency of mitotic defects such as chromosome misalignments, multipolar spindles, lagging chromosomes, chromatin bridges, and micronuclei (Fig. 1b, c, and Supplementary Movie 1–4). We also found that USP35-depleted cells failed cytokinesis at significantly higher rates than control cells (Fig. 1d, e, and Supplementary Movie 5 and 6). These phenotypes caused by USP35 deficiency were confirmed again by analyzing fixed cells with immunostained microtubules. Three or more daughter cells were formed, or bi- or multi-nuclear phenotypes were exhibited in USP35-deficient cells at the late telophase. These phenotypes are evidence of cytokinesis failure^15^. More than 30% of USP35-deleted cells were bi- or multi-nucleates compared to approximately 10% of control cells after cytokinesis (Fig. 1f). In addition, spindle multi-polarity occurred frequently in USP35-deficient mitotic cells (Fig. 1g and Supplementary Fig. 1c). Since there have been several studies reporting how the formation of multipolar spindles results from abnormal conditions including cytokinesis failure and mitotic slippage^16^, we were able to anticipate that USP35 is associated with faithful mitosis progress and cytokinesis. From these data, we could anticipate that USP35 is associated with faithful mitosis progression and cytokinesis. The defects observed in USP35-deficient cells were largely rescued by the expression of WT USP35, but not by the expression of the catalytically inactive form of USP35, USP35^C450A^ (Fig. 1g and Supplementary Fig. 1c). The same results were observed when a different siRNA (USP35i-#2) targeting USP35 was transfected (Supplementary Fig. 1d, e), which means that the USP35 DUB activity is important in mitotic progression.

### USP35 interacts with and deubiquitinates Aurora B

**Fig. 2:**
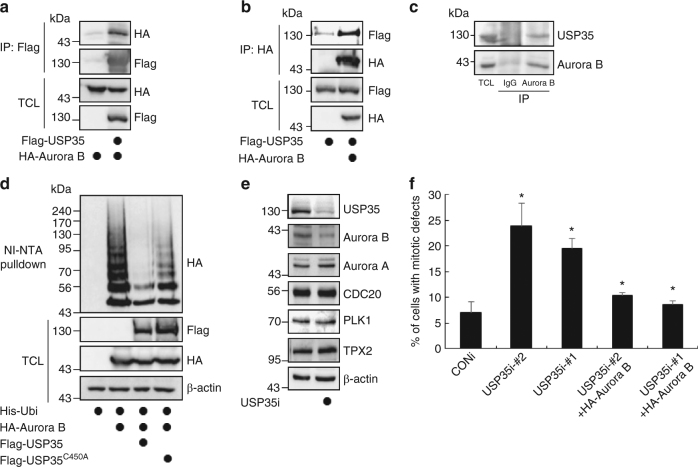
USP35 is a DUB for Aurora B. **a** HEK293T cells were transfected with HA-Aurora B alone or in combination with Flag-USP35. The interaction between Flag-USP35 and HA-Aurora B was detected by immunoblotting after immunoprecipitation with an anti-Flag antibody. TCL, total cell lysates. **b** HEK293T cells were transfected with Flag-USP35 alone or in combination with HA-Aurora B. The interaction between Flag-USP35 and HA-Aurora B was detected by immunoblotting after immunoprecipitation with an anti-HA antibody. **c** The interaction between endogenous USP35 and Aurora B was detected by immunoblotting after immunoprecipitation with an anti-Aurora B antibody. **d** HEK293T cells transfected with His-ubiquitin alone or in combination with HA-Aurora B, Flag-USP35 or Flag-USP35^C450A^ were synchronized by a treatment with 100 ng/mL nocodazole (NOC) for 18 h and then treated with the proteasome inhibitor MG132 for 4 h. Aurora B ubiquitination was observed using a Ni-NTA-mediated pulldown assay. **e** HeLa cells transfected with CONi or USP35i were synchronized in prometaphase by a treatment with 100 ng/mL NOC for 18 h. The cell lysates were immunoblotted using the indicated antibodies. **f** HeLa cells were transfected with USP35i-#1 or USP35i-#2 alone or in combination with HA-Aurora B. Cells showing several defects were counted after immunofluorescence staining with β-tubulin Cy3 antibody. One-hundred cells per group were examined from three independent experiments. The data in a part **f** represent the mean ± SD (*P < 0.05; **P < 0.005, *t*-test)

We wanted to delineate the molecular mechanism by which the depletion of USP35 increases the frequency of mitotic defects and cytokinesis failures. Aurora B kinase is a key regulator of mitosis since it is involved in all stages of the mitotic process including prometaphase chromosomal congregation, metaphase chromosomal alignment, anaphase chromosomal segregation, and the completion of cytokinesis^7^. When the Aurora B function is disrupted by either RNA interference or inhibitors, mitotic slippage results from the abolishment of error correction or checkpoint satisfaction, and cytokinesis failure typically occurs during mitosis^17–19^. These phenotypes observed during Aurora B inhibition are similar to those caused by USP35 deficiency. Hence, we questioned whether the effect of USP35 on the regulation of mitosis was mediated in an Aurora B-dependent manner. To test this possibility, we investigated the interaction between USP35 and Aurora B. When we transfected HEK293T cells with Flag-USP35 and HA-Aurora B, we were able to observe the binding of USP35 to Aurora B by co-immunoprecipitation (Fig. 2a, b). Consistent with this, ectopically expressed Flag-USP35 was associated with endogenous Aurora B in HEK293T and HeLa cells, and conversely, endogenous USP35 was precipitated efficiently by HA-Aurora B affinity purification (Supplementary Fig. 2a, b, c). In addition, USP35 interacted with Aurora B in physiological condition (Fig. 2c). These results showed that USP35 interacts with Aurora B. Next, we examined whether USP35 interacts with other CPC proteins. However, we could see no interaction between other endogenous CPC proteins, such as INCENP or Survivin, and Flag-USP35 by immunoprecipitation with an anti-Flag antibody (Supplementary Fig. 2d). From these results, we concluded that USP35 specifically interact with Aurora B in the CPC proteins. Next, we tested whether USP35 could deubiquitinate Aurora B kinase. As shown in Fig. 2d, Aurora B was ubiquitinated in the presence of ubiquitin, and deubiquitinated by WT USP35, but not by USP35^C450A^. Furthermore, the deubiquitination of Aurora B was promoted by USP35 but not by other USPs, such as USP15 or USP44 (Supplementary Fig. 2e, f). These data suggest that USP35 is a candidate DUB for Aurora B.

### USP35 regulates the levels of Aurora B protein

Since it is known that the ubiquitination of Aurora B regulates its stability or localization^8,9^, we then tested whether USP35 could perturb the protein levels or localization of Aurora B. When the expression of USP35 was decreased by siRNA, the levels of Aurora B protein were also reduced in nocodazole-treated HeLa cells (Fig. 2e), and the localization of Aurora B was mostly similar between USP35-depleted cells and control cells during mitosis. However, in the metaphase, we could not clearly verify its localization because the Aurora B signal intensity was significantly diminished by the USP35 knockdown, compared to the control siRNA treatment (Supplementary Fig. 3a). These results suggest that USP35-mediated deubiquitination affects the protein levels of Aurora B, but not its localization. To confirm whether USP35 knockdown induces the formation of multipolar spindles by reducing the levels of Aurora B protein, we transfected HeLa cells with siRNA targeting USP35 alone or in combination with HA-Aurora B plasmid. Consistent with Fig. 1f, Supplementary Fig. 1d, e, USP35-depleted cells exhibited a high-frequency of mitotic defects, whereas Aurora B overexpression rescued these errors induced by USP35 knockdown (Fig. 2f, Supplementary Fig. 3b, c). Therefore, these data suggest that USP35 is involved in mitotic progression by regulating the protein levels of Aurora B by deubiquitination.

### USP35 inhibits CDH1-mediated degradation of Aurora B

**Fig. 3:**
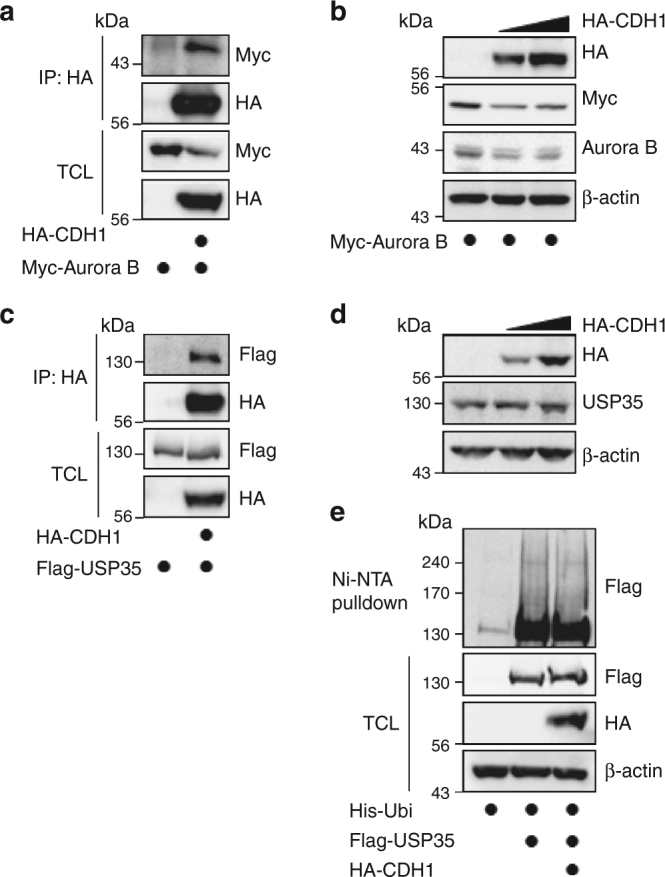
CDH1 interacts with both USP35 and Aurora B, but has no effects on USP35. **a** HEK293T cells were transfected with Myc-Aurora B alone or in combination with HA-CDH1. The interaction between HA-CDH1 and Myc-Aurora B was detected by immunoblotting after immunoprecipitation with an anti-HA antibody. **b** HEK293T cells were transfected with Myc-Aurora B alone or in combination with two different concentrations of HA-CDH1. A western blot analysis was performed to detect Aurora B protein levels. **c** HEK293T cells were transfected with Flag-USP35 alone or in combination with HA-CDH1. The interaction between HA-CDH1 and Flag-USP35 was detected by immunoblotting after immunoprecipitation with an anti-HA antibody. **d** A western blot analysis was conducted to detect USP35 protein levels in lysates from HEK293T cells transfected with two different concentrations of HA-CDH1. **e** HEK293T cells were transfected with His-ubiquitin alone or in combination with Flag-USP35 or HA-CDH1 and then treated with MG132 for 4 h. USP35 ubiquitination was observed using a Ni-NTA-mediated pulldown assay

Having noted the effect of USP35 depletion on the levels of Aurora B protein, we expected that USP35 could deubiquitinate Aurora B, and counteract the ubiquitination activity of APC^CDH1^ E3 ligase. Aurora B is ubiquitinated by APC when it has been activated by CDH1^20^. As reported previously, we were able to observe the interaction between CDH1 and Aurora B and to confirm that the co-expression of CDH1 caused a dose-dependent decrease in the levels of Aurora B protein (Fig. 3a, b). Interestingly, we also observed that USP35 could bind to CDH1 (Fig. 3c). To exclude the possibility that CDH1 directly controls USP35 levels, we undertook an ubiquitination assay of USP35 and checked the USP35 protein levels in the presence of CDH1. However, CDH1 had no effect on the levels of USP35 protein and could not induce the ubiquitination of USP35 (Fig. 3d, e).

**Fig. 4:**
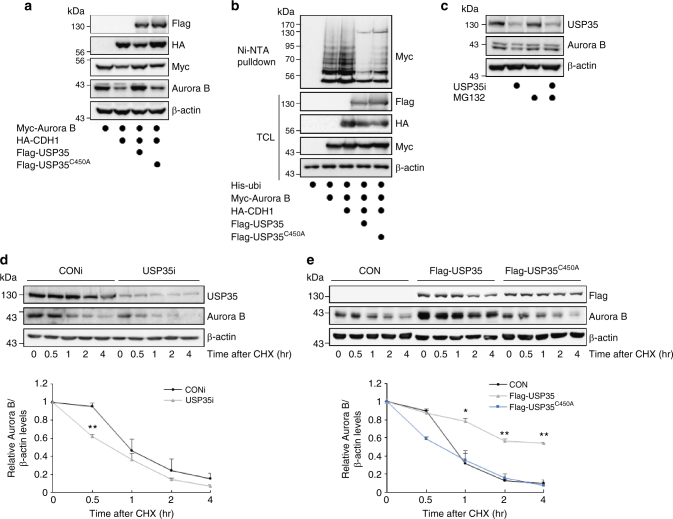
USP35 maintains Aurora B stability by blocking APC^CDH1^-mediated proteasomal degradation. **a** HEK293T cells were transfected with Myc-Aurora B alone or in combination with HA-CDH1, Flag-USP35, or Flag-USP35^C450A^. The cell lysates were immunoblotted using the indicated antibodies. **b** HEK293T cells transfected with His-ubiquitin alone or in combination with Myc-Aurora B, HA-CDH1, Flag-USP35, or Flag-USP35^C450A^ were synchronized in prometaphase by treatment with 100 ng/mL NOC for 18 h and then treated with MG132 for 4 h. Aurora B ubiquitination was observed using a Ni-NTA-mediated pulldown assay. **c** HeLa cells transfected with CONi or USP35i were synchronized in prometaphase by a treatment with 100 ng/mL NOC for 18 h and then treated with MG132 for 6 h prior to harvesting. A western blot analysis was conducted to detect Aurora B protein levels. **d** HeLa cells transfected with CONi or USP35i were synchronized in prometaphase by a treatment with 100 ng/mL NOC for 18 h. The cells were then treated with 100 μg/mL cycloheximide (CHX) and harvested at the times indicated. A western blot analysis was conducted to detect Aurora B protein levels. Quantification of Aurora B levels was done considering the amount of β-actin protein in each case. **e** HeLa cells were transfected with Flag-USP35 or Flag-USP35^C450A^ and then treated with 100 μg/mL CHX. The cells were harvested at the times indicated. A western blot analysis was performed to detect Aurora B protein levels. Quantification of Aurora B levels was done considering the amount of β-actin protein in each case. The data in parts **d** and **e** from three independent experiments represent the mean ± SD (*P < 0.05, **P < 0.005, *t*-test)

E3 ligases and their counteracting DUBs often bind to each other, which allows the reversible modification of common substrates^21^. As expected, the CDH1-mediated reduction in the levels of Aurora B protein was recovered by the expression of WT USP35, but not by the expression of USP35^C450A^ (Fig. 4a). We also examined the influence of USP35 on the ubiquitination of Aurora B. CDH1 triggered an increase in the ubiquitination of Aurora B, and USP35 could deubiquitinate Aurora B in the presence of CDH1 (Fig. 4b). Indeed, USP35 deficiency did not affect the protein levels of APC^CDH1^ targets other than Aurora B (Fig. 2e). These results support the contention that USP35 counteracts the APC^CDH1^-mediated ubiquitination of Aurora B, but USP35 per se may not be affected by APC^CDH1^. To investigate whether CDC20, which is another APC activator, regulates Aurora B ubiquitination or not, we examined the interaction between Aurora B and CDC20. However, CDC20 did not bind to Aurora B, and did not alter its protein levels (Supplementary Fig. 4a, b). In addition, CDC20 had no effects on Aurora B ubiquitination (Supplementary Fig. 4c). When we transfected synchronized prometaphase cells with siRNA targeting USP35 only, the Aurora B level was decreased. The reduced Aurora B level by USP35 knockdown was rescued by the co-depletion of USP35 and CDH1 but not by co-depletion of USP35 and CDC20. (Supplementary Fig. 4d). These data indicate that CDC20 is not the relevant APC/C adaptor for Aurora B degradation.

APC^CDH1^ triggers 26S-mediated proteasomal degradation of substrates by assembling K11-linked ubiquitin chains^22^. Thus, USP35 should remove the polyubiquitins at the K11 residue of ubiquitin-attached Aurora B to prevent Aurora B degradation. To identify whether USP35 could cleave K11-linked ubiquitin chains in substrates, we performed a deubiquitination assay using recombinant K11-, K48-, or K63-linked tetra-ubiquitin chains. As determined by a western blot analysis, USP35 could mainly disassemble K11- and K63-conjugated ubiquitin chains, and it could also weakly disassemble K48-linked chains (Supplementary Fig. 5). These results indicate that USP35 has no specificity for ubiquitin chains and can prevent the degradation of Aurora B modified with K11-linked ubiquitin chains by APC^CDH1^. In line with this result, USP35 knockdown did not reduce the levels of Aurora B protein when we treated prometaphase-arrested HeLa cells with the proteasome inhibitor MG132 (Fig. 4c). For a further investigation of USP35-induced changes in the levels of Aurora B protein, we treated HeLa cells that were synchronized at the prometaphase with cycloheximide and harvested the cells for a time-course analysis. The depletion of USP35 led to more rapid degradation of Aurora B than that observed in control cells (Fig. 4d). Conversely, the stability of Aurora B was maintained by WT USP35, but not by USP35^C450A^, despite the cycloheximide treatment (Fig. 4e). Altogether, these data indicate that USP35 maintains Aurora B stability by blocking APC^CDH1^-induced proteasomal degradation.

### USP35 affects Aurora B functions in mitosis

**Fig. 5:**
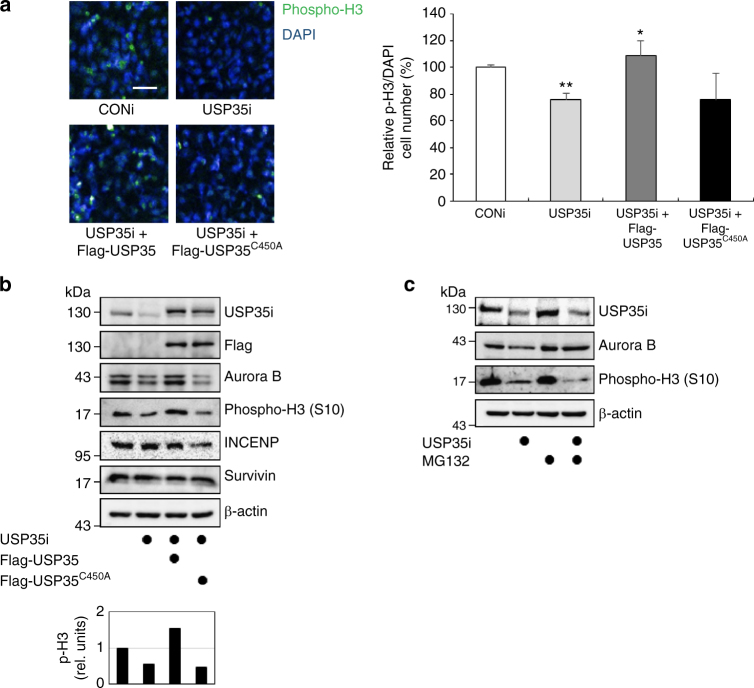
USP35 regulates Aurora B functions in mitosis. **a** HeLa cells were transfected with USP35i alone or in combination with Flag-USP35 or Flag-USP35^C450A^. The cells were stained with a phospho-histone H3 (Ser10) antibody (left). The cells expressing green fluorescence (phospho-H3, phospho-histone H3) were counted and normalized to DAPI staining (right). At least 4000 cells per group were examined from three independent experiments. Scale bar = 50 μm. **b** HeLa cells transfected with USP35i alone or in combination with Flag-USP35 or Flag-USP35^C450A^ were synchronized by a treatment with 100 ng/mL NOC for 18 h. The cell lysates were immunoblotted using the indicated antibodies. Quantification of the phospho-histone H3 (p-H3) levels was done considering the amount of β-actin protein in each case. **c** HeLa cells transfected with CONi or USP35i were synchronized in prometaphase by treatment with 100 ng/mL NOC for 18 h and then treated with MG132 for 4 h prior to harvesting. A western blot analysis was utilized to detect Aurora B and phospho-histone H3 (Ser10) protein levels. The data in part **a** represent the mean ± SD (*P < 0.05, **P < 0.005, *t*-test)

Previous studies have reported that Aurora B-depleted cells display a significant reduction in the levels of phospho-histone H3, which is a substrate of Aurora B^7^. Thus, we questioned whether USP35 could regulate the functions of Aurora B as a kinase, and we used the phosphorylation of histone H3 as an indicator of Aurora B downstream signaling. By immunofluorescence staining, we were able to observe a reduction of histone H3 phosphorylation in USP35-depleted cells compared to that in control cells (Fig. 5a). Western blot analysis also showed that the depletion of USP35 lowered the levels of both phospho-histone H3 and Aurora B protein in nocodazole-treated cells. These levels were restored by the expression of WT USP35, but not by the expression of USP35^C450A^ (Fig. 5b), suggesting that USP35-mediated deubiquitination modulates Aurora B stability, consequently altering its function. To identify whether a decrease in phospho-histone H3 levels could be caused by reduced Aurora B levels in USP35-deficient cells, we observed Aurora B levels and phospho-histone H3 levels in MG132-arrested metaphase cells. As shown in Fig. 4c, USP35 knockdown reduced the Aurora B protein level in nocodazole-arrested cells, but not in MG132-arrested cells. However, phospho-histone H3 levels were decreased by USP35 knockdown regardless of whether MG132 was present or not (Fig. 5c). These data suggest that reduced Aurora B protein levels in USP35-depeleted cells do not cause a decrease in H3 phosphorylation and thus that USP35 regulates not only Aurora B protein levels but also Aurora B activity. Indeed, USP35 knockdown could not disrupt the levels of other CPC proteins, such as INCENP or Survivin (Fig. 5b), indicating that USP35 does not affect other CPC proteins and may only have an effect on Aurora B. Based on these data, we concluded that USP35-induced deubiquitination of Aurora B could affect its downstream signaling, which is required for faithful mitotic progression.

### FoxM1 is a transcription factor for USP35 expression

**Fig. 6:**
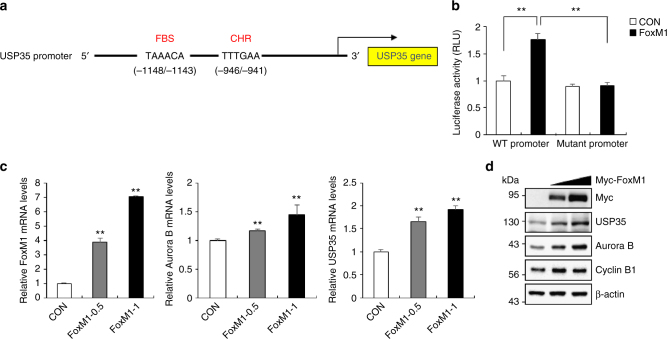
USP35 is a FoxM1 target gene. **a** A schematic of the promoter region of the human USP35 gene. The sequences and positions of a FoxM1-binding site (FBS) and a cell cycle genes homology region (CHR) in the USP35 promoter are shown. **b** HEK293T cells were transfected with vectors that expressed the luciferase reporter gene under the control of either the WT USP35 promoter or a mutant USP35 promoter, either alone or in combination with Myc-FoxM1. The luciferase activity was measured and normalized to the renilla luciferase activity. **c**, **d** HeLa cells transfected with Myc-FoxM1 were synchronized in prometaphase by a treatment with 100 ng/mL NOC for 18 h. **c** A q-PCR analysis was conducted to determine the relative expression levels of mRNAs encoding *USP35*, *FoxM1*, and *Aurora B*, and **d** a western blot analysis was performed to detect USP35 and FoxM1 target protein levels. The data in parts **b** and **c** represent the mean ± SD (*P < 0.05; **P < 0.005, *t*-test; *n* *=* 3)

The expression levels of most cell cycle regulators fluctuate throughout the cell cycle. Since USP35 could function during mitotic progression, a part of the cell cycle, via an Aurora B-dependent pathway, we then considered that USP35 expression may also be regulated during the cell cycle and that this would be similar to that of Aurora B. This hypothesis prompted us to examine whether the transcription factor FoxM1 was involved in USP35 expression. FoxM1 is a critical transcription factor for the expression of mitotic regulators such as Cyclin B, PLK1, and Aurora B during the G2/M phase^23^. We further identified a FoxM1-binding sequence (FBS; 5′-TAAACA-3′) and a cell cycle gene homology region (CHR; 5′-TTTGAA-3′), which are required for FoxM1 binding to the promoter of target genes^24^, in the USP35 promoter region (Fig. 6a). We cloned this promoter region into the pGL3-luciferase reporter plasmid to generate one plasmid expressing WT FBS and WT CHR, and another expressing a mutant FBS (5′-TAACCA-3′) and WT CHR. A luciferase assay in HeLa cells transfected with these luciferase reporter plasmids and Myc-FoxM1 showed that FoxM1 enhanced the activity of the WT promoter but not that of the mutant promoter (Fig. 6b). In addition, the mRNA and protein levels of both USP35 and Aurora B were increased by FoxM1 in a dose-dependent manner (Fig. 6c, d). These findings demonstrate that USP35 expression is regulated by a FoxM1-dependent pathway during the cell cycle.

## Discussion

We showed that the depletion of USP35 inhibited metaphase chromosome alignment, which generated lagging chromatin during the chromosome segregation process. In addition, USP35-depleted cells exhibited an increased number of multipolar spindles compared to control cells. At the late anaphase, chromatin bridges were detected in USP35-depleted cells. These conditions cause a failure to complete cytokinesis and result in bi- or multi-nucleated cells^25^. The phenotype of USP35-depleted cells is strikingly similar to that of cells with defective CPC proteins, in particular, with defective Aurora B. Aurora B, a Ser/Thr kinase is a subunit of a CPC that contains three other proteins: INCENP, Survivin, and Borealin. During mitosis, this complex plays several roles that correlate with the diverse functions of Aurora B, which include modifying histones, correcting erroneous kinetochore-microtubule attachments, and regulating cytokinesis^7^.

A number of papers have reported that the ubiquitination of Aurora B is important for the regulation of its dynamic localization and for the correct timing of its degradation and that it thus tightly controls the function of Aurora B during mitosis^26,27^. The stability of Aurora B is under the control of the APC/C E3 ligase pathway. APC activated by CDH1 recognizes KEN-box, D-box, and/or A-box sequences of Aurora B and degrades Aurora B via the ubiquitin-proteasomal system at the end of mitosis^8,20^. Meanwhile, the localization of Aurora B is affected by another E3 ligase, a Cul3-based E3 ligase. Similar to the APC/C activation system, the BTB adaptors form a complex with Cul3 and facilitate Cul3 activation, thus regulating the dynamic localization of Aurora B on mitotic chromosomes and its accumulation at the spindle midzone after anaphase onset. Aurora B directly binds to these BTB adaptors and is modified via Cul3-mediated ubiquitination^27,28^. Moreover, it has recently been reported that UBASH3B acts as an ubiquitin receptor for Aurora B ubiquitinated by Cul3 and drives the recruitment of Aurora B to mitotic microtubules^29^.

Here, we provide evidences related Aurora B deubiquitination. USP35 can bind to CDH1 and restore Aurora B levels that have been reduced by CDH1-induced ubiquitination, suggesting that USP35 counteracts the APC^CDH1^-mediated ubiquitination of Aurora B. This possibility supports the occurrence of mitotic defects in USP35-depleted cells, as dysregulated levels of Aurora B protein may interfere with the progression of mitosis. Indeed, the APC^CDH1^-mediated ubiquitination of Aurora B is known to occur at the end of mitosis. Thus, the levels of Aurora B protein are not affected by CDH1 during early mitosis. There is a possibility that Aurora B ubiquitination is actually performed by APC^CDC20^, which is known to have basal activity in early mitosis. However, we could not find any relationship between Aurora B and CDC20 (Supplementary Fig. 4). We surmise that Aurora B forms a complex with USP35 and CDH1 during mitosis, whereas USP35 mostly maintains Aurora B levels because the CDK1-dependent ubiquitination of CDH1 inhibits interaction with APC/C in the early phase. At the end of mitosis, APC E3 ligase activated by CDH1 acts on Aurora B ubiquitination much more strongly than USP35, thus degrading Aurora B. In order to support this hypothesis, further study is required to identify the mechanism that regulates USP35 activity during mitosis.

The APC/C E3 ligase functions with two different E2 enzymes, UBE2C, and UBE2S, to assemble K11 linkages during mitosis. Specifically, UBE2S elongates the K11-ubiquitin chain initiated by another E2 enzyme UBCH10 for CDH1-dependent anaphase substrates^30,31^. Because Aurora B is also an APC/C substrate activated by CDH1, it has UBE2S-assembled K11-linked ubiquitin chains and is degraded by 26S proteasome. USP35 showed differences in catalytic efficiency but did not have strict ubiquitin chain-type specificity, like most USPs^32^. Thus, USP35 can hydrolyze K11-linked polyubiquitin chains of Aurora B assembled by the APC^CDH1^ complex. However, we could find no evidence that USP35 modifies the Cul3-mediated ubiquitination of Aurora B reversibly (Supplementary Fig. 6). Perhaps the DUB activity of USP35 is applied differently depending on the E3 ligase, despite the fact that it is the same substrate. The difference in USP35 activity with respect to E3 ligases could be explained if different E3 ligases modify different lysine residues of Aurora B. Indeed, multiple lysines in the N-terminus of Aurora B are required for its degradation, whereas only K56 is important for the correct localization of Aurora B by Cul3 E3 ligase^29,33^. Another explanation could be an additional post-translational modification on Aurora B for USP35 activity. Phosphorylation by CDKs and PLKs as well as autophosphorylation is essential to activate Aurora B. Also, relocalization of Aurora B during anaphase requires to remove CDK1-mediated inhibitory phosphorylation^7^. Hence, phosphorylation of Aurora B is likely to be necessary for USP35 activity.

Earlier, Lindon et al.^26^ reported that Aurora B localization could be altered by APC^CDH1^. According to their study, Aurora B strongly localized to a diffuse punctate band in the region of the equatorial cortex, but not to the spindle midzone, in CDH1-depleted early anaphase cells. However, during the late anaphase and telophase, Aurora B localized to the cleavage furrow in both CDH1-depleted cells and control cells, suggesting that APC^CDH1^ was required for Aurora B localization in the spindle midzone at the early anaphase. We observed that the Aurora B signal intensity was significantly decreased in USP35-depleted metaphase cells, but not in cells in other phases. This observation suggests an additional function of USP35; i.e., it may, partially regulate Aurora B localization during metaphase.

In conclusion, we have found that during mitosis Aurora B can be regulated by USP35. The depletion of USP35-induced mitotic defects by decreasing the stability of Aurora B and its downstream signaling. Deubiquitination by USP35 protects Aurora B from APC^CDH1^-induced degradation, allowing proper chromosome segregation. Collectively, our data suggest that USP35 functions as a mitotic regulator that promotes high-fidelity mitotic progression.

## Methods

### Plasmids and siRNAs

A p3XFlag-CMV^TM^7.1-hUSP35 was provided from GenScript. The catalytic inactive USP35 mutant (USP35^C450A^) was generated by PCR-based site-directed mutagenesis using p3XFlag-CMV^TM^7.1-hUSP35 as a template. Myc- and HA-Aurora B clones were kinds of gifts from Prof. Chang-woo Lee (Sungkyunkwan Univ.). Ubiquitin and other DNAs were cloned into pCS2-His, -HA, or -Myc vectors for expression in mammalian cells. Control siRNA and siRNA targeting *USP35*, *CDH1*, or *CDC20* were synthesized from Bioneer. siRNA sequences are as follows: *USP35*-#1, 5′-GGGAAGATCTGATGATGTT-3′; *USP35*-#2, 5′-CCAAGAGGAAGGATGGTAC-3′; *CDH1*, 5’- TGAGAAGTCTCCCAGTCAGAA-3’; *CDC20*, 5′- GGAGCTCATCTCAGGCCATAA-3′

### Cell culture and transfection

HEK293T cells and HeLa cells purchased from Korea Cell Line Bank (KCLB) were cultured in Dulbecco’s modified Eagle medium (DMEM) containing 10% fetal bovine serum and 1% penicillin and streptomycin and maintained at 37 °C in 5% CO_2_. All the cell lines used in this study have been authenticated by KCLB and confirmed to be free of mycoplasma contamination prior to use. For transient transfection, HEK293T cells were transfected with plasmids using 2 M CaCl_2._ and 2 × HBS buffer (50 mM HEPES, 10 mM KCl, 12 mM Glucose, 280 mM NaCl, 1.5 mM Na_2_HPO_4,_ pH 7.05) and HeLa cells were transfected with plasmids or siRNAs using Lipofectamine^TM^ 2000 (Invitrogen) following the manufacturer’s instructions.

### Time-lapse microscopy

Time-lapse live imaging was performed using the 40 × , 1.35NA 0.10 mm WD objective lens on a microscope (DeltaVision Cpre; GE Healthcare) equipped with a charge-coupled device (CCD) camera (photometrics) on CO_2_ chamber at 37 °C (Applied Precision). HeLa cells stably expressing histone H2B-GFP were a kind of gift from Dr. Toru Hirota (The Cancer Institute, Japanese Foundation for Cancer Research)^34^. The cells were seeded onto a glass-bottom dish (8-well chamber, Lab-Tek; II Chambered Coverglass, Thermo Fisher) and transfected USP35 siRNA or control siRNA. Time-lapse images were taken at 3 min intervals as three sections with 5-μm z-steps using 40 × , 1.35NA 0.10 mm WD objective lens and maximally projected. All data were obtained from three independent experiments and all samples were blinded before the analysis.

### Western blot analysis

Western blot analyses on 10–50 μg protein extracts from HeLa cells or HEK293T cells. Briefly, we lysed the cells using protein lysis buffer (50 mM Tris-Cl, 150 mM NaCl, 1% Triton X-100, 1 mM EDTA, 200 mM Na_3_VO_4_, 1 × proteinase inhibitor, pH 7.4), and measured the protein concentration using Micro BCA^TM^ protein Assay kit (Thermo Scientific) based on the standard curve using BSA. The following antibodies were used for immunoblot analysis; rabbit anti-USP35 (A302-290A, dilution ratio 1:1000), rabbit anti-Aurora B (A300-431A, 1:1000), and rabbit anti-Aurora A (A300-071A, 1:1000) were purchased from Bethyl Laboratories. Mouse anti-Aurora B (ab3609, 1:1000) and rabbit anti-PLK1 (ab47073, 1:1000) were purchased from Abcam. Mouse anti-HA (sc-7392, 1:1000), mouse anti-c-Myc (sc-40, 1:1000), mouse anti-Survivin (sc-17779, 1:500), mouse anti-INCENP (sc-376514, 1:1000), rabbit anti-CDC20 (p55 CDC) (sc-8358, 1:1000), rabbit anti-Cyclin B1 (sc-594, 1:1000), and goat anti-Mad2 (sc-6329, 1:1000) were provided from Santa Cruz Biotechnology. Rabbit anti-TPX2 (NB500-179, 1:1000) was purchased from Novus Biologicals. Rabbit anti-phospho-histone H3 (Ser10) (#06-570, 1:1000) was obtained from Millipore. Mouse anti-Flag (F1804, 1:1000) was purchased from Sigma Aldrich and rabbit anti-Ubiquitin (#3933, 1:2000) was purchased from Cell signaling. Mouse anti-HSP90α/β (sc-13119, Santa Cruz Biotechnology, 1:5000), and rabbit anti-β-actin (LF-PA0207, AbFrontier, 1:5000) were used to assess equal loading. Samples were analyzed by sodium dodecyl sulfate polyacrylamide gel electrophoresis (SDS-PAGE) and western blot and chemiluminescence was measured using the Ez-Capture MG imaging system (ATTO Corporation). Full blot images used in this study are provided in Supplementary Fig. 7.

### Immunoprecipitation

HEK293T cells transfected with various plasmids as indicated for 24 h were collected and lysed using protein lysis buffer (50 mM Tris-HCl, 150 mM NaCl, 1% Triton X-100, 1 mM EDTA, 200 mM Na_3_VO_4_, 1 × proteinase inhibitor, pH 7.5). About 3 mg of lysates were incubated with Flag-agarose beads (Sigma Aldrich) or HA-agarose beads (Sigma Aldrich) for 4 h at 4 °C. Immunocomplexes were washed with lysis buffer, and eluted and boiled in 6 × SDS sample buffer. For fully endogenous immunoprecipitation, about 5 mg of lysates from HEK293T cells were incubated with rabbit IgG (sc-2027, Santa Cruz Biotechnology, 1 μg/1 mg lysates) or rabbit anti-Aurora B (A300-431A, Bethyl Laboratories, 1 μg/1 mg lysates) for 4 h at 4 °C. After incubation, protein A agarose beads (Genedepot) were added and incubated again for 12 h at 4 °C. Immunocomplexes were washed with lysis buffer, and eluted and boiled in 6 × SDS sample buffer. All samples were detected by western blot analysis using the indicated antibodies, and 5 % of the samples were used to identify immunoprecipitation efficiency. Full blot images used in this study are provided in Supplementary Fig. 7.

### Ni-NTA-mediated pulldown assay

HEK293T cells transfected with His-ubiquitin and tagged plasmids as indicated were synchronized by treatment with 100 ng/mL nocodazole for 18 h followed by treatment with 10 μM MG132 (A.G. Scientific. Inc.) for 4 h. The cells were lysed with Urea lysis buffer (8 M Urea, 0.3 M NaCl, 50 mM Na_2_HPO_4,_ 50 mM Tris-HCl, 1 mM phenylmethylsulfonyl fluoride (PMSF), 10 mM Imidazole, pH 8) and sonicated. About 3–5 mg of lysates were incubated with Ni-NTA agarose (Qiagen) for 6 h at 4 °C. The beads were washed with Urea washing buffer (8 M Urea, 0.3 M NaCl, 50 mM Na_2_HPO_4,_ 50 mM Tris-HCl, 1 mM PMSF, 20 mM Imidazole, pH 8) and eluted in 6 × SDS sample buffer. The samples were detected by western blot analysis. Full blot images used in this study are provided in Supplementary Fig. 7.

### Immunofluorescence

HeLa cells transfected with USP35-targeting siRNA alone or in combination with WT USP35 or USP35^C450A^ were grown on glass coverslips. The cells were fixed with cold methanol for 15 min on ice and permeabilized with 1 × phosphate buffered saline (PBS) containing 0.25 % Triton X-100. After washing, the cells were incubated with rabbit anti-TPX2 (1:1000), rabbit anti-Aurora B (1:1000), or rabbit anti-phospho-histone H3 (Ser10) (1:500) for 2 h at room temperature, followed by Alexa fluor-488-conjugated goat anti-rabbit IgG (Molecular Probes, 1:300) for 1 h at room temperature. Microtubules were stained with anti-β-tubulin-Cy3 antibody (C4585, Sigma Aldrich, 1:100) and DNA was detected with DAPI (Sigma Aldrich). In experiments identified the spindle checkpoint bypass, HeLa cells were seeded in the 35 mm confocal dish and transfected with USP35-targeting siRNA or control siRNA. After 48 h, the cells were treated with 100 nM Taxol (Sigma Aldrich) for 24 h and then added 20 μM Hoechst (Molecular Probes) to detect DNA. Four percent formaldehyde was used to fix the cells into the media. The cells stained with anti-Aurora B, anti-TPX2 antibodies, or Hoechst were visualized using 40 × or 100 × magnification on an LSM700 Confocal Laser Scanning Microscopy (Carl-Zeiss) and analyzed using ZEN 2012 imaging software (Carl-Zeiss). The fluorescence response of phospho-histone H3 staining cells was automatically imaged using Operetta^®^ High Contents Image System (PerkinElmer^TM^) and quantified using Harmony 3.1 software (PerkinElmer^TM^). All data were obtained from three independent experiments and all samples were blinded before the analysis.

### Deubiquitination assay

The DUB activity of USP35 was tested using recombinant K11-, K48-, and K63-linked ubiquitin tetramers (Boston Biochem)^12^. HeLa cells transfected with p3XFlag-CMV^TM^7.1 vector or 3 × Flag-USP35 were collected and lysed using protein lysis buffer. About 3 mg of lysates were incubated with Flag-agarose for 4 h at 4 °C. The beads were washed with lysis buffer and eluted in DUB buffer (20 mM HEPES at pH 7.4, 100 mM NaCl) with Flag peptide for 30 min at 4 °C. The eluted samples and recombinant K11-, K48-, or K63-linked ubiquitin tetramers were mixed with 5 mM dithiothreitol (DTT) and reacted for 1 h at 30 °C. These reactions were stopped by adding 6 × SDS sample buffer. The samples were detected by western blot analysis using an anti-ubiquitin antibody.

### Quantitative reverse transcription-PCR analysis

Total RNA from cells was extracted using RNeasy Mini Kit (Qiagen). cDNAs were synthesized using ReverTra Ace^®^ q-PCR RT Master Mix (Toyobo) and analyzed using SYBR^®^ Green Real-time PCR Master Mix (Toyobo) on a CFX Connect^TM^ Real-Time PCR (Bio-Rad). Primers were designed using OligoPerfect^TM^ Designer (Invitrogen). All data were normalized to β-actin expression. Primer sequences are as follows: *USP35*-F, TCGAATCTGTCAGCAACGTC; *USP35*-R, TGTCTTTGGAAATGGCTTCC; *Aurora B*-F, GGGAGAGCTGAAGATTGCTG; *Aurora B*-R, GCACCACAGATCCACCTTCT; *FoxM1*-F, TCTCGGAGGAAACAGCATCT, *FoxM1*-R, CAGAGGAGTCTGCTGGGAAC; *β-actin*-F, CTCTTCCAGCCTTCCTTCCT; *β-actin*-R, AGCACTGTGTTGGCGTACAG

### Luciferase assay

To measure the USP35 promoter activity, we amplified the −1260/+269 regions (containing FBS and CHR) of the human USP35 promoter from BAC clone (#RPCI-11; 480O10, Empire Genomics) by PCR using the primers containing *Xho*1 and *Hin*dIII restriction sites, respectively. This fragment was cloned into a pGL3-basic firefly luciferase reporter vector after digestion with *Xho*1 and *Hin*dIII enzymes (NEB). PCR-based site-directed mutagenesis was performed to generate a single point mutation in a FoxM1 binding site (5'-TAAACA-3' → 5'-TAACCA-3') of USP35 promoter region using PrimeSTAR^®^ HS Premix (Takara). The sequences of these vectors were verified by DNA sequencing. HEK293T cells were transfected with pRL-renilla luciferase reporter vector and WT USP35 promoter vector or its mutant vector or in combination with Myc-FoxM1. The luciferase assays were performed using Dual-Glo^®^ Luciferase Reagent (Promega) on a SpectraMax^®^ M3 (Molecular Devices). All data were normalized to renilla luciferase activity.

### Statistical analysis

Results are shown as mean ± SD of at least three independent experiments, unless otherwise indicated in the figure legends. The comparison of different groups was carried out using two-tailed unpaired Student’s *t*-test, and the *P*-value < 0.05 was considered statistically significant and reported as in legends.

### Data availability

All relevant data are available from the corresponding author upon reasonable request.

## Electronic supplementary material

Supplementary Information

Peer Review File

Description of Additional Supplementary Files

Supplementary Movie 1

Supplementary Movie 2

Supplementary Movie 3

Supplementary Movie 4

Supplementary Movie 5

Supplementary Movie 6
