# Supplementary material for: USP35 regulates mitotic progression by modulating the stability of Aurora B
Source: Nat Commun. 2018 Feb 15;9:688. doi: 10.1038/s41467-018-03107-0 (PMC5814453; doi:10.1038/s41467-018-03107-0)
Supplement: Supplementary file 1 — Supplementary Information [file 41467_2018_3107_MOESM1_ESM.docx]

**
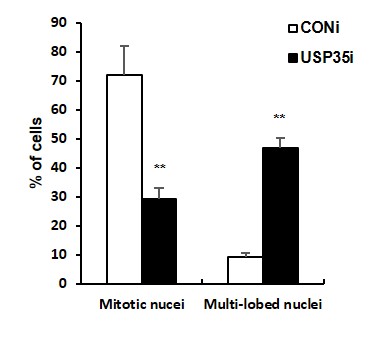

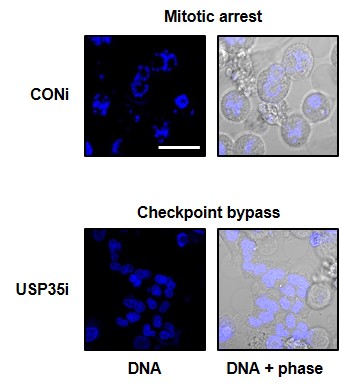
a**

**
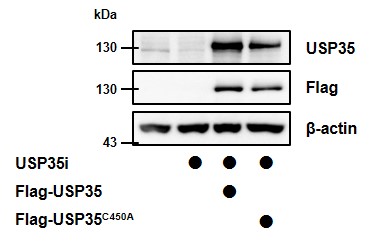

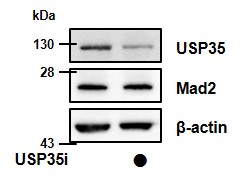
b** **c**

**
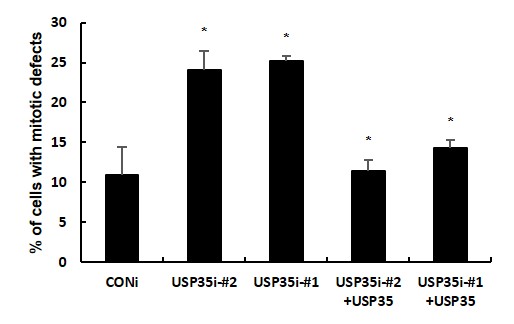
d** **e**

**
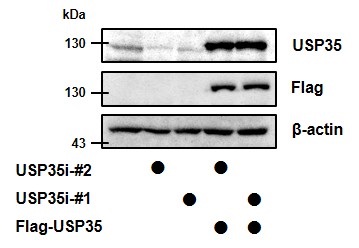
**

**Supplementary Figure 1. The depletion of USP35 leads to mitotic checkpoint bypass in Taxol-treated cells.**

**(a**) HeLa cells transfected with control siRNA (CONi) or siRNA targeting USP35 (USP35i) were treated with Taxol for 24 hr. Nuclei were stained with Hoechst (Left). The cells with nuclear abnormalities were counted after nuclear staining (Right). 100 cells per group were examined from three independent experiments. Scale = 20 μm .**(b)** Using cell lysates from the experiments that generated the data shown in Figure 1a-e, western blot analysis was performed to check the efficiency of USP35i. Mad2 was used as a marker of siRNA toxicity. **(c)** Representative western blot analysis from the experiments summarized in Figure 1g. **(d)** HeLa cells were transfected with USP35i-#1 or USP35i-#2 alone or in combination with Flag-USP35. Cells showing several defects were counted after immunofluorescence staining with β-tubulin Cy3 antibody. 100 cells per group were examined from three independent experiments. **(e)** Representative western blot analysis from (d) experiments. The data in parts **a** and **d** represent the mean ± SD (**P*<0.05; ***P*<0.005, *t*-test).

**
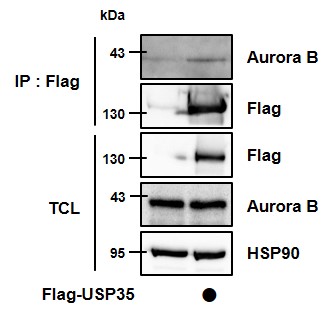
a** **b**

**
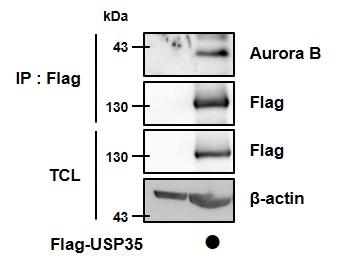
**

**
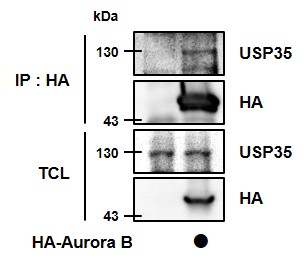

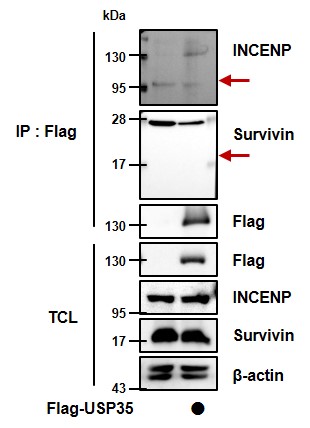
c**  **d**

**
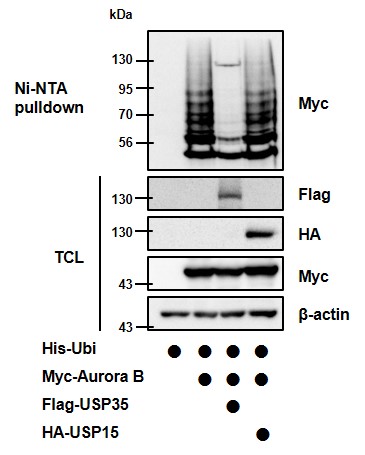

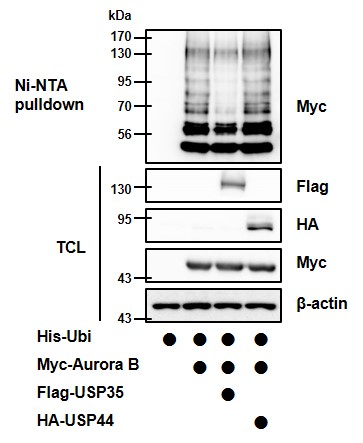
e** **f**

**Supplementary Figure 2. USP35 is a potential DUB for Aurora B.**

**(a**) 293T cells were transfected with control or Flag-USP35 plasmids. The interaction between Flag-USP35 and endogenous Aurora B was detected by immunoblotting after immunoprecipitation with an anti-Flag antibody. **(b)** HeLa cells were transfected with control or Flag-USP35 plasmids. The interaction between Flag-USP35 and endogenous Aurora B was detected by immunoblotting after immunoprecipitation with an anti-Flag antibody. **(c)** 293T cells were transfected with control or HA-Aurora B plasmids. The interaction between HA-Aurora B and endogenous USP35 was detected by immunoblotting after immunoprecipitation with an anti-HA antibody. **(d)** 293T cells were transfected with control or Flag-USP35 plasmids. The interaction between Flag-USP35 and endogenous INCENP or Survivin was detected by immunoblotting after immunoprecipitation with an anti-Flag antibody. **(e)** 293T cells transfected with His-ubiquitin alone or in combination with Myc-Aurora B, Flag-USP35, or HA-USP15 were synchronized by a treatment with NOC, and then treated with MG132 for 4 hr. Aurora B ubiquitination was observed using a Ni-NTA-mediated pulldown assay. **(f)** HeLa cells transfected with His-ubiquitin alone or in combination with Myc-Aurora B, Flag-USP35, or HA-USP44 were synchronized by a treatment with NOC, and then treated with MG132 for 4 hr. Aurora B ubiquitination was observed using a Ni-NTA-mediated pulldown assay.

**
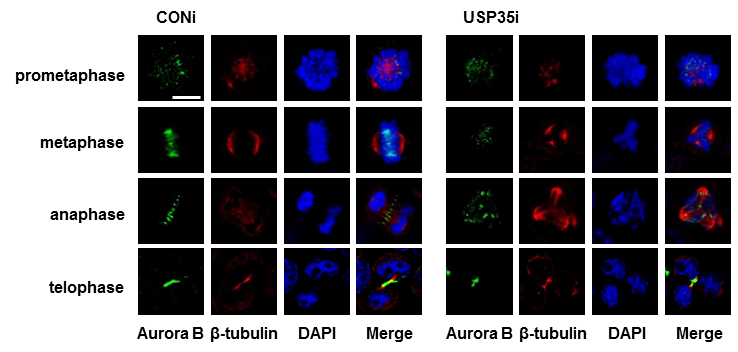
a**

**
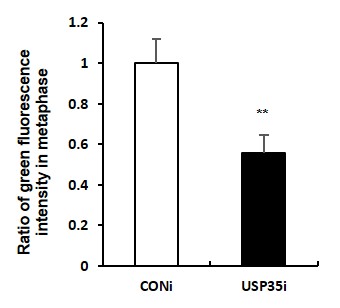
**

**b c**

**
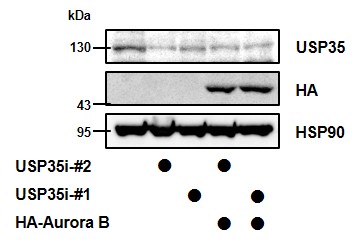

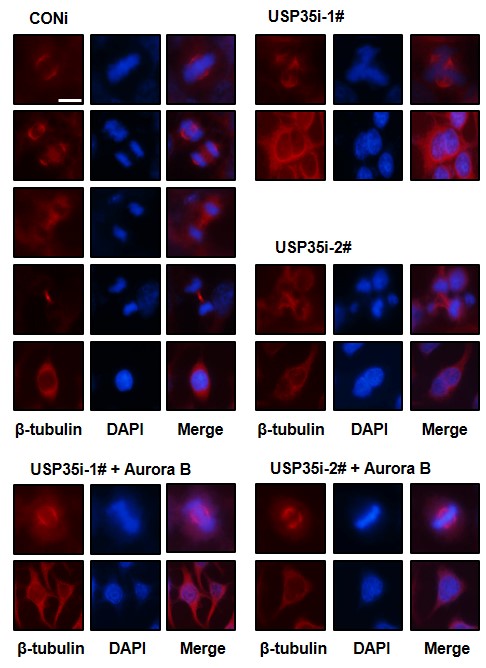
**

**Supplementary Figure 3. The localization of Aurora B is not altered by USP35 knockdown.**

**(a**) HeLa cells transfected with CONi or USP35i were stained with antibodies against Aurora B and β-tubulin (Left). The Aurora B signal intensity in the metaphase was analyzed by a NIS-Elements software (Right). 100 cells per group were examined from three independent experiments. Scale bar = 10 μm. **(b** and **c) (b)** Representative staining images and **(c)** western blot analysis from the experiments summarized in Figure 2f. Scale bar = 10 μm. The data in a part **a** represent the mean ± SD (**P*<0.05; ***P*<0.005, *t*-test)

**
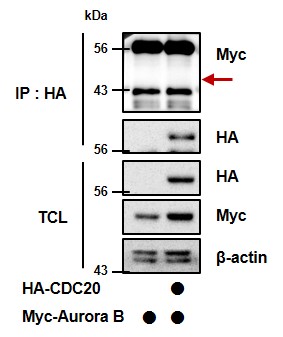
a** **b**

**
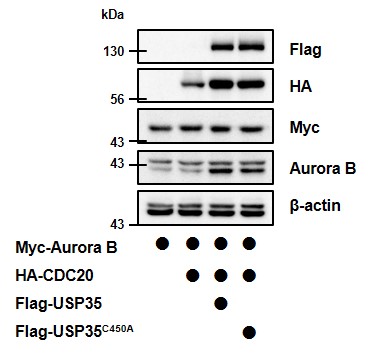
**

**
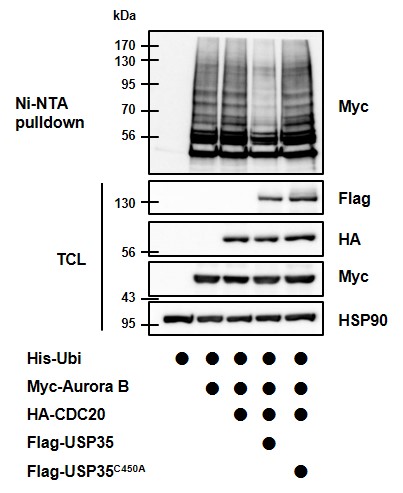
c** **d**

**
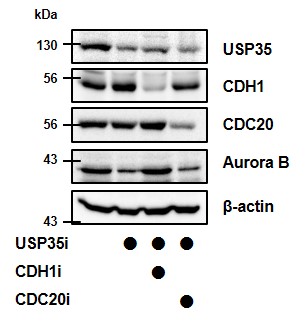
**

**Supplementary Figure 4. CDC20 has no effect on Aurora B.**

**(a)** 293T cells were transfected with Myc-Aurora B alone or in combination with HA-CDC20. The interaction between HA-CDC20 and Myc-Aurora B was detected by immunoblotting after immunoprecipitation with an anti-HA antibody. **(b)** 293T cells were transfected with Myc-Aurora B alone or in combination with HA-CDC20, Flag-USP35 or Flag-USP35C450A. The cell lysates were immunoblotted using the indicated antibodies. **(c)** 293T cells transfected with His-ubiquitin alone or in combination with Myc-Aurora B, HA-CDC20, Flag-USP35 or Flag-USP35^C450A^ were synchronized in the prometaphase by a treatment with 100 ng/mL NOC for 18 hr and then treated with MG132 for 4 hr. Aurora B ubiquitination was observed using a Ni-NTA-mediated pulldown assay. **(d)** HeLa cells transfected with USP35i alone, or in combination with siRNA targeting *CDH1* (CDH1i) or *CDC20* (CDC20i) were synchronized in the prometaphase by a treatment with 100 ng/ml NOC for 18 hr. Western blot analysis was performed to detect Aurora B protein levels.


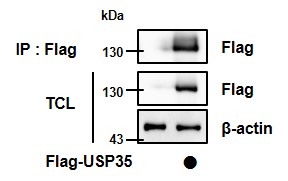


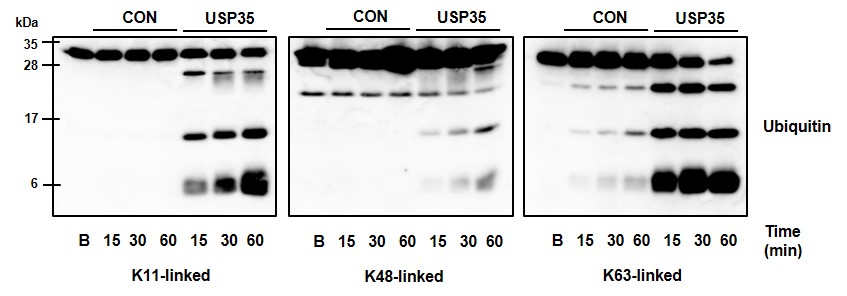


**Supplementary Figure 5. USP35 DUB activity.**

HeLa cells were transfected with Flag or Flag-USP35. The cell lysates were incubated with K11-, K48-, or K63-linked ubiquitin tetramers for the indicated times after immunoprecipitation with an anti-Flag antibody. Each cleaved ubiquitin tetramer was detected by immunoblotting with an anti-ubiquitin antibody. B, only buffer


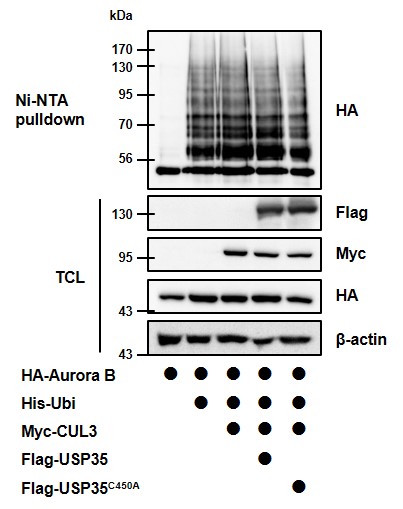


**Supplementary Figure 6. USP35 has no effect on CUL3-mediated ubiquitination of Aurora B.**

293T cells transfected with HA-Aurora B alone or in combination with His-Ubiquitin, Myc-CUL3, Flag-USP35 or Flag-USP35^C450A^ were synchronized in the prometaphase by a treatment with 100 ng/mL NOC for 18 hr and then treated with MG132 for 4 hr. Aurora B ubiquitination was observed using a Ni-NTA-mediated pulldown assay.

**Supplementary Fig. 7**


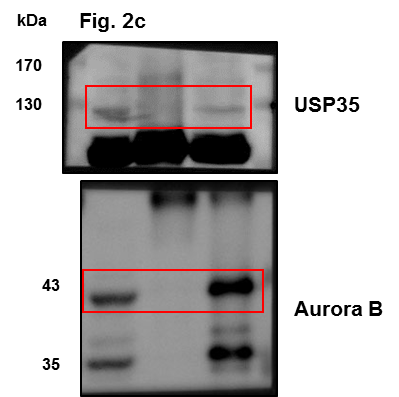

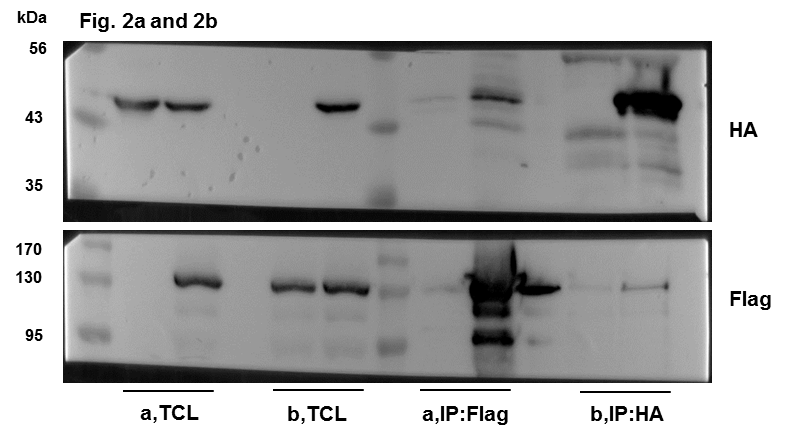


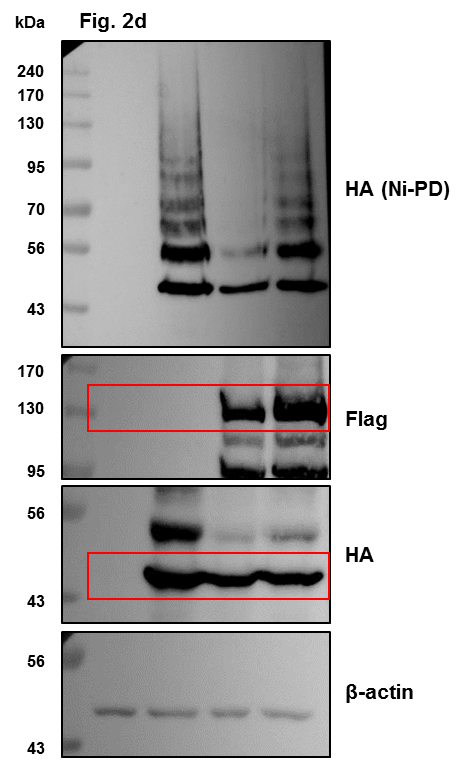

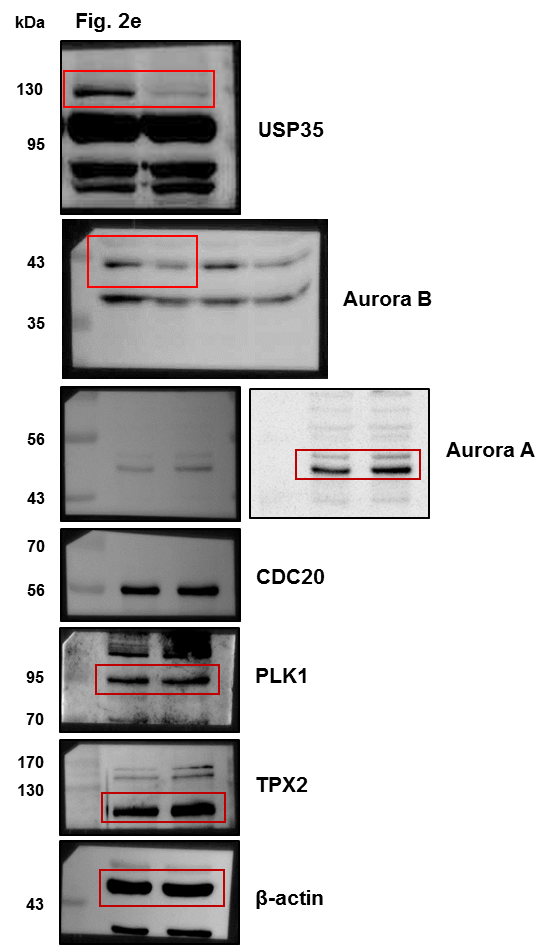


**Supplementary Fig. 7**

**
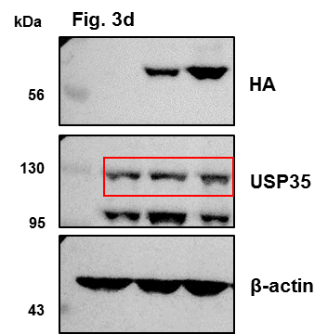

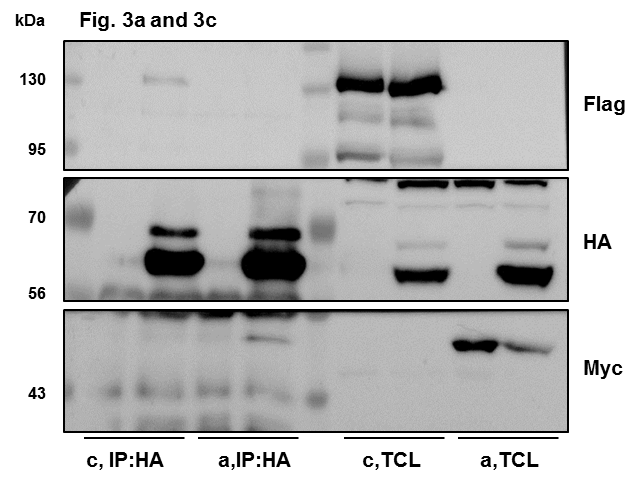
**

**
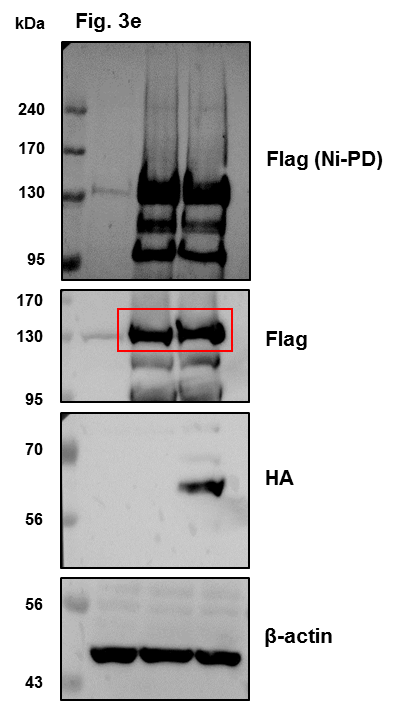

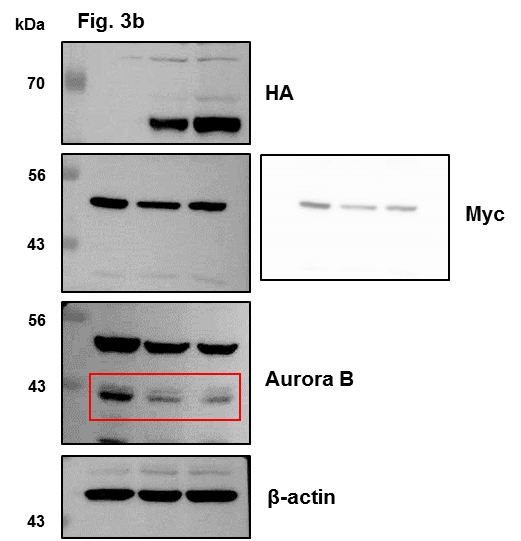
**

**Supplementary Fig. 7**

**
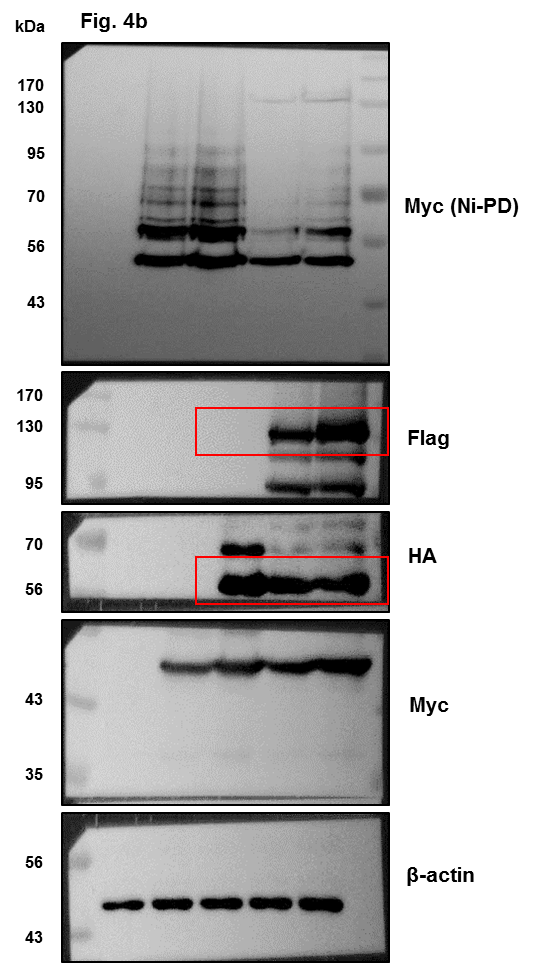

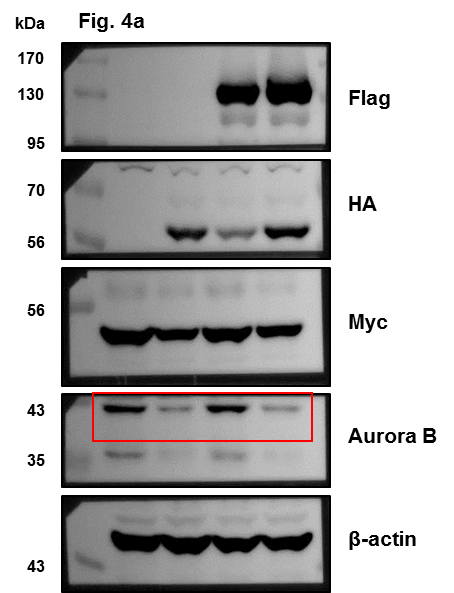
**

**
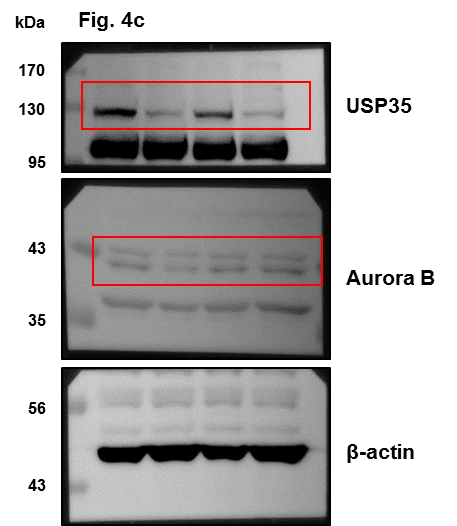
**

**
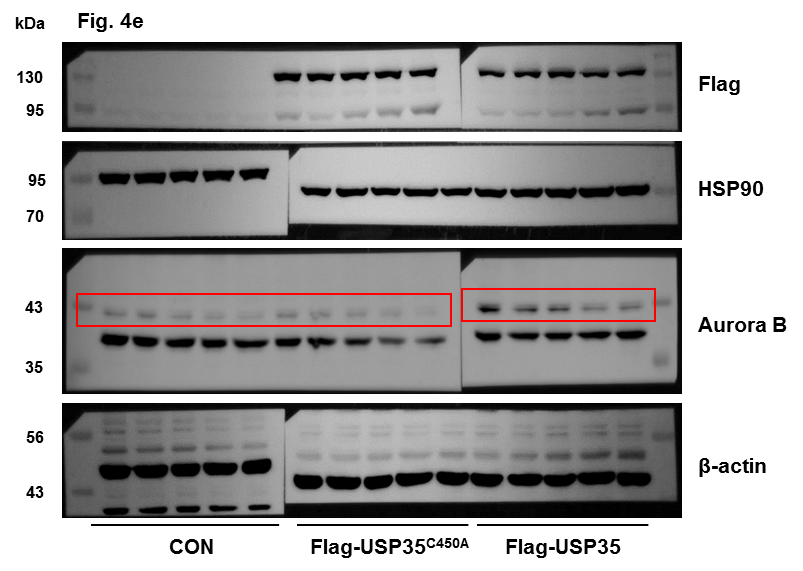
**

**Supplementary Fig. 7**

**
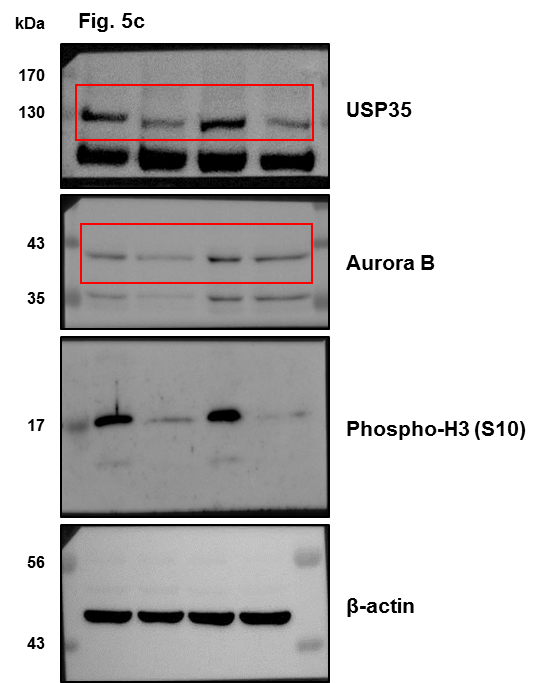

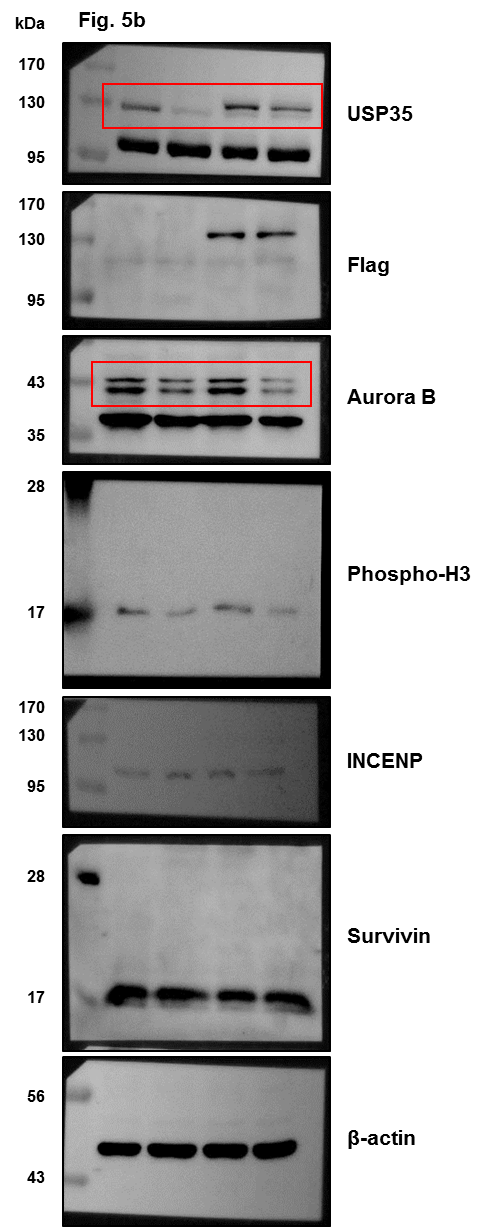
**

**
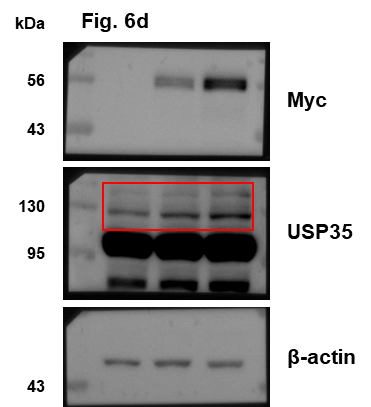
**

**Supplementary Fig. 7**

**
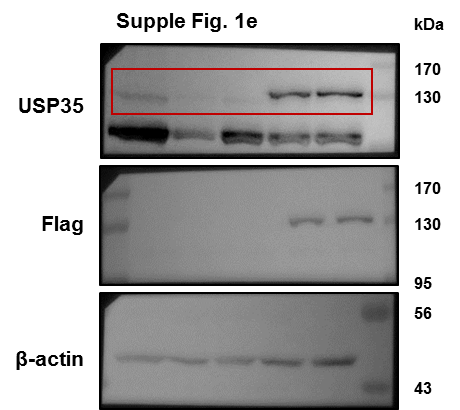

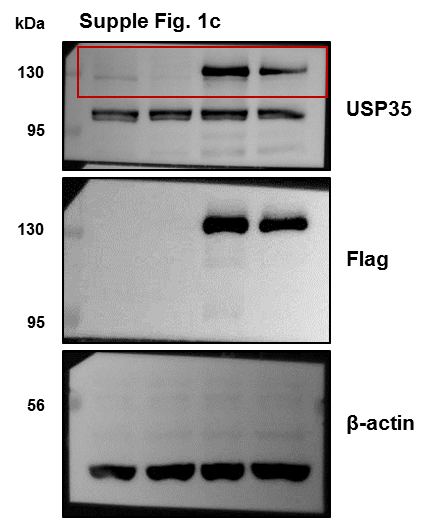

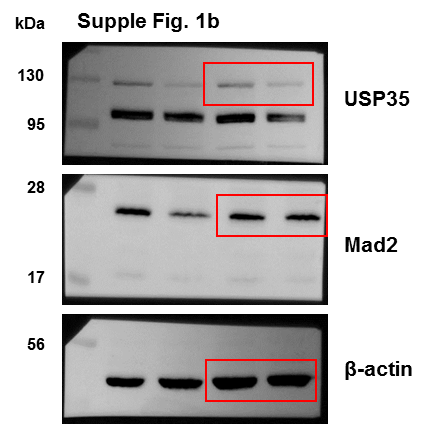
**

**
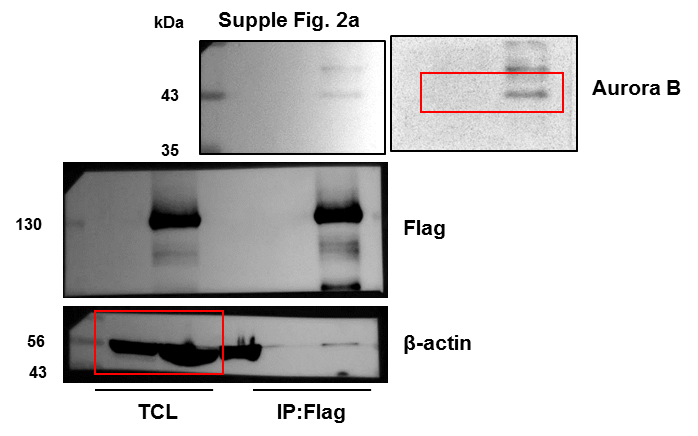

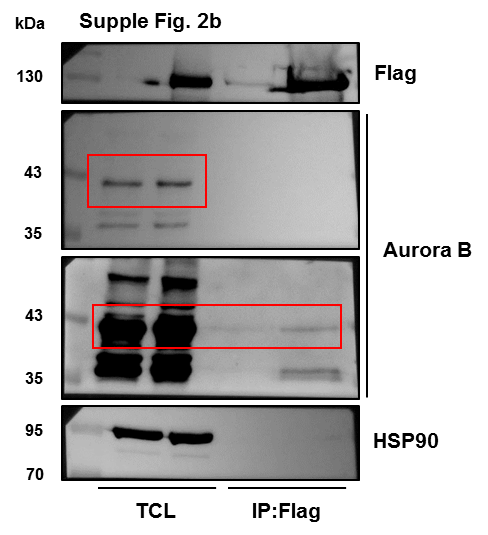
**

**
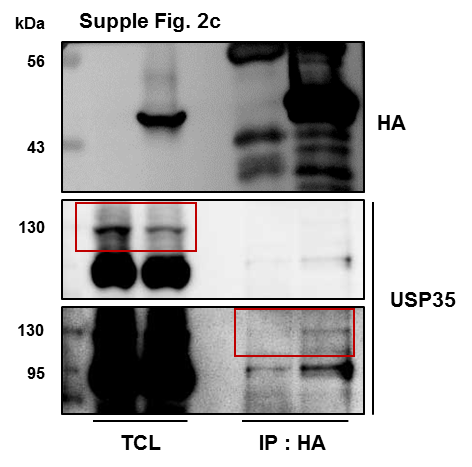

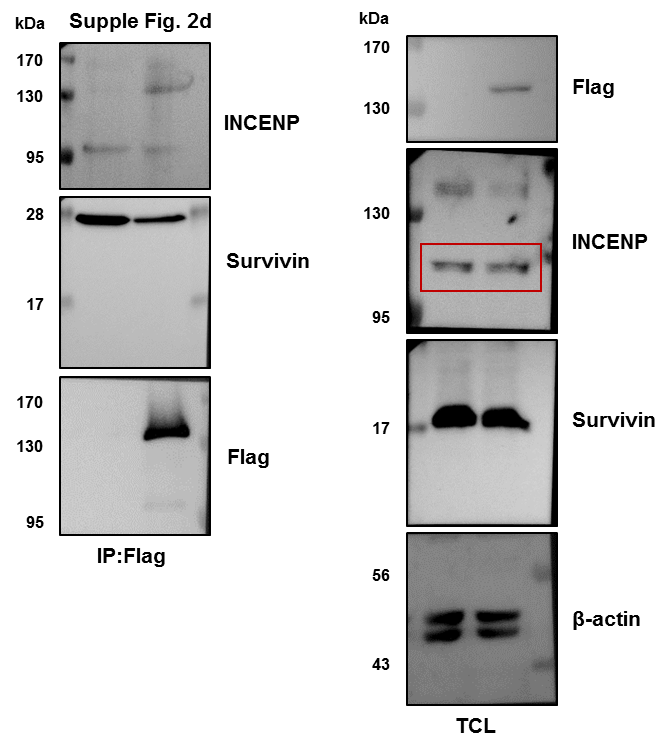
**

**Supplementary Fig. 7**

**
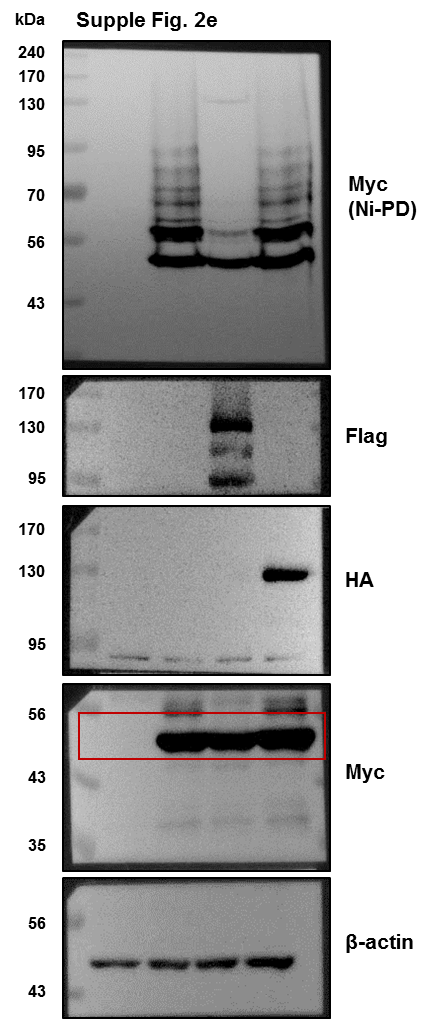
**

**
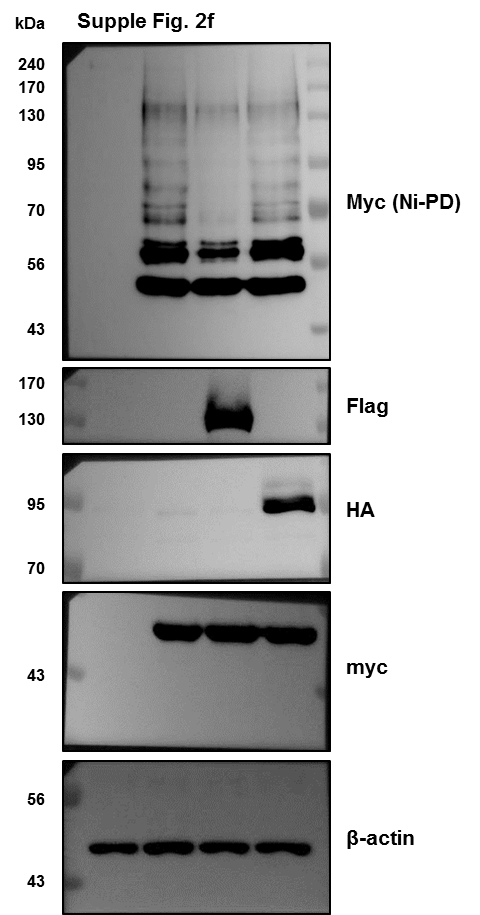
**

**
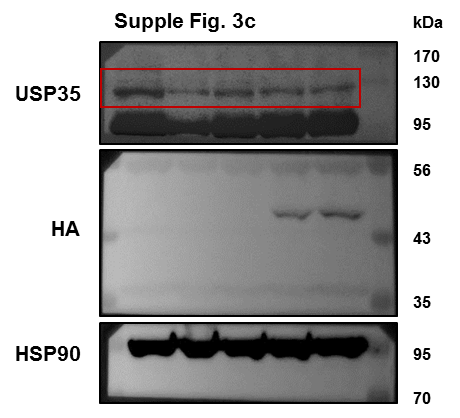
**

**Supplementary Fig. 7**

**
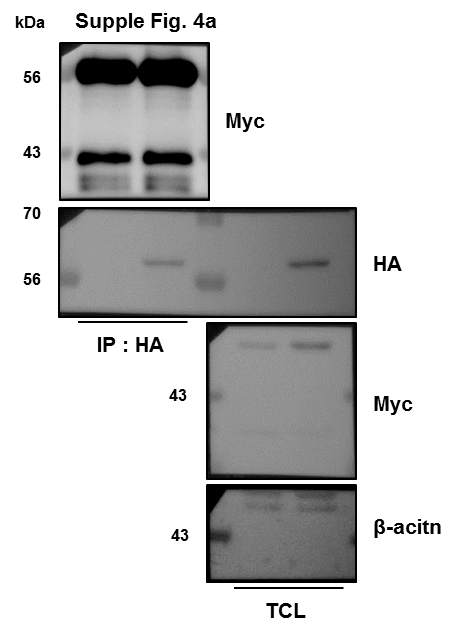

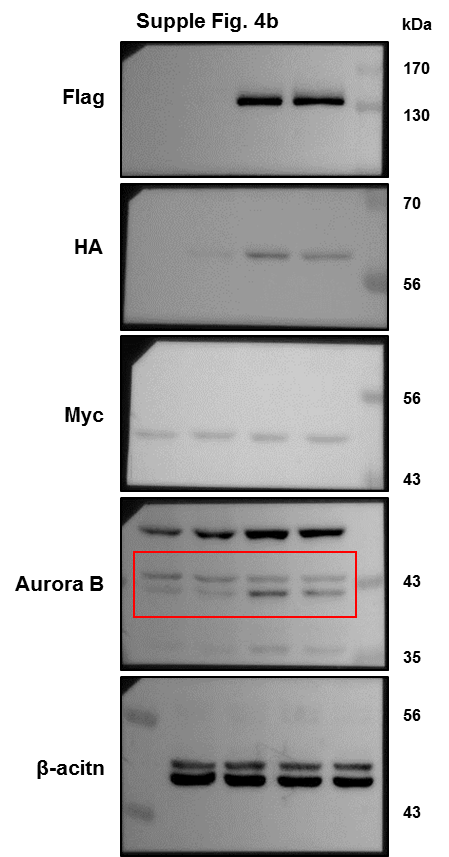
**

**
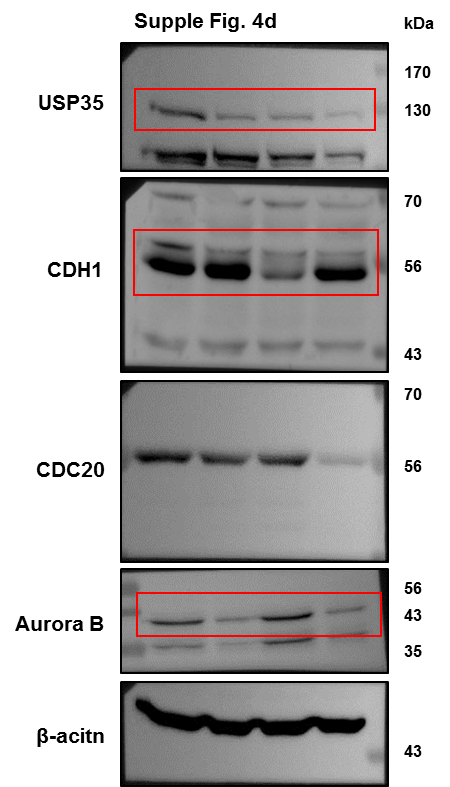

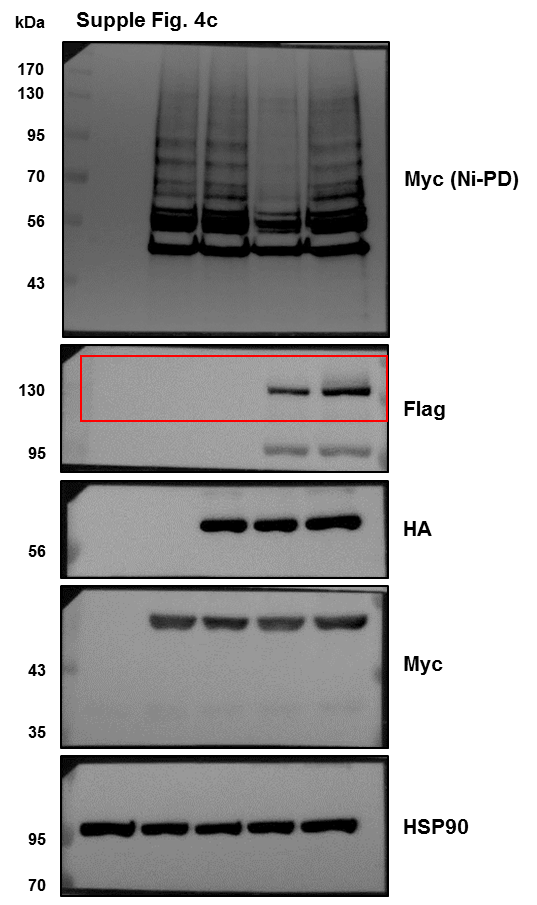
**

**Supplementary Fig. 7**

**
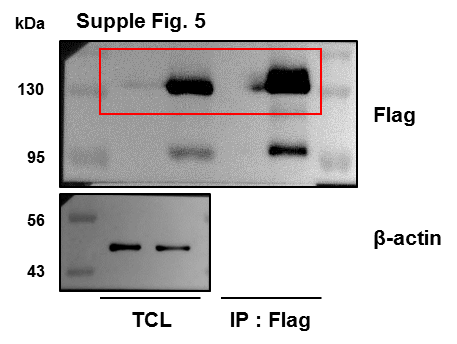
**

**
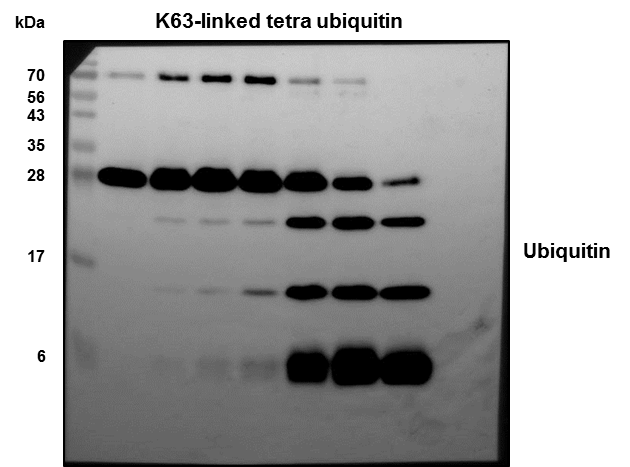

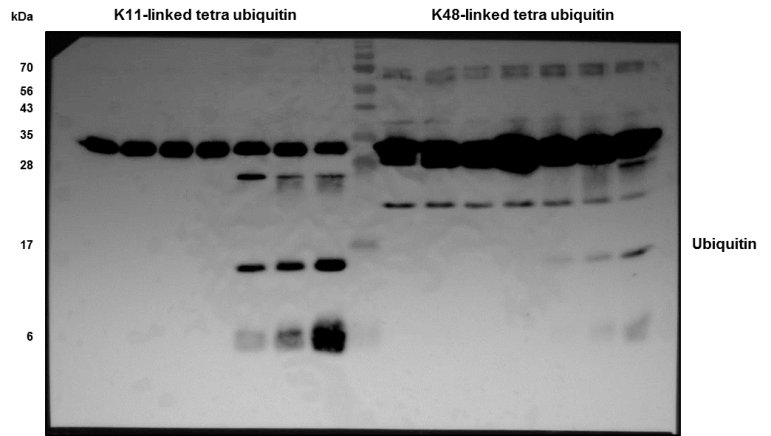
**

**
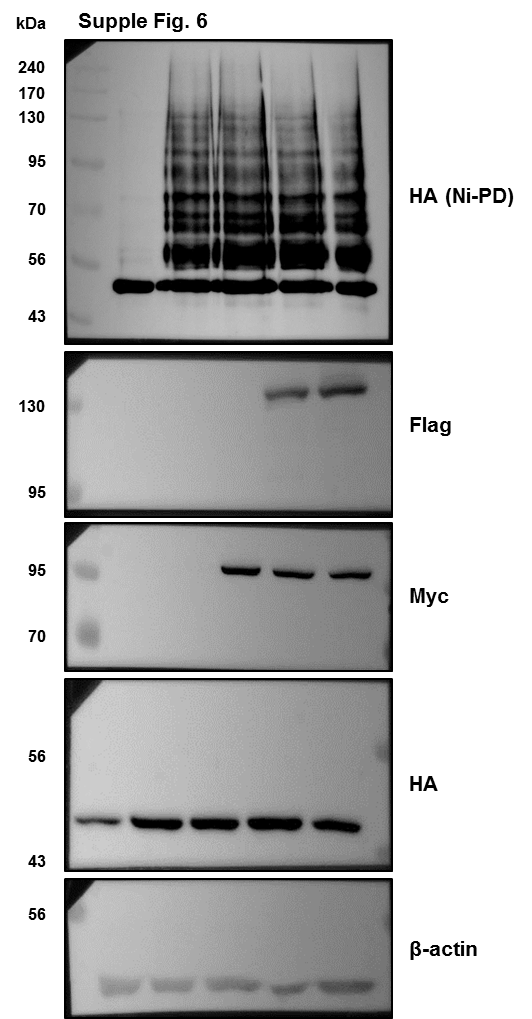
**

**Supplementary Figure 7. Full western blot images used in this study.**
