# Supplementary material for: USP35 regulates mitotic progression by modulating the stability of Aurora B
Source: Nat Commun. 2018 Feb 15;9:688. doi: 10.1038/s41467-018-03107-0 (PMC5814453; doi:10.1038/s41467-018-03107-0)
Supplement: Supplementary file 3 — Description of Additional Supplementary Files [file 41467_2018_3107_MOESM3_ESM.pdf]

Description of Additional Supplementary Files

File Name: Supplementary Movie 1
Description: Mitosis in control HeLa cells. GFP-H2B expressing HeLa cells were transfected with control siRNA (CONi) and then treated with thymidine. GFP-H2B was used to visualize chromosome movement. This video shows normal mitosis. The images were captured every 3 min and they are played back at 5 frames per second. Scale bar = 10 μm.

File Name: Supplementary Movie 2
Description: Mitosis in USP35-depleted HeLa cells. GFP-H2B expressing HeLa cells were transfected with siRNA targeting *USP35* (USP35i) and then treated with thymidine. This video shows the presence of misaligned chromosomes and mitotic delay during mitosis. The images were captured every 3 min and they are played back at 5 frames per second. Scale bar = 10 μm.

File Name: Supplementary Movie 3
Description: Mitosis in USP35-depleted HeLa cells. GFP-H2B expressing HeLa cells were transfected with USP35i and then treated with thymidine. This video shows the presence of misaligned chromosomes, chromatin bridges, improperly separated chromosomes, and micronucleus during mitosis. The images were captured every 3 min and they are played back at 5 frames per second. Scale bar = 10 μm.

File Name: Supplementary Movie 4
Description: Mitosis in USP35-depleted HeLa cells. GFP-H2B expressing HeLa cells were transfected with USP35i and then treated with thymidine. This video shows the presence of anaphase lagging chromosomes during mitosis. The images were captured every 3 min and they are played back at 5 frames per second. Scale bar = 10 μm.

File Name: Supplementary Movie 5
Description: Cytokinesis in control HeLa cells. GFP-H2B expressing HeLa cells were transfected with CONi and then treated with thymidine. This video shows normal mitosis and cytokinesis. The images were captured every 3 min and they are played back at 10 frames per second. DNA is shown in green (GFP-H2B). Scale bar = 10 μm.

File Name: Supplementary Movie 6
Description: Cytokinesis in USP35-depleted HeLa cells. GFP-H2B expressing HeLa cells were transfected with USP35i and then treated with thymidine. This video shows abnormal mitosis and cytokinesis failures. The images were captured every 3 min and they are played back at 10 frames per second. DNA is shown in green (GFP-H2B). Scale bar = 10 μm.
