# Supplementary material for: USP35 regulates mitotic progression by modulating the stability of Aurora B
Source: Nat Commun. 2018 Feb 15;9:688. doi: 10.1038/s41467-018-03107-0 (PMC5814453; doi:10.1038/s41467-018-03107-0)
Supplement: Supplementary file 2 — Peer Review File [file 41467_2018_3107_MOESM2_ESM.pdf]

Response to Reviewers' Comments

We would like to thank reviewers and the editor for the valuable comments and suggestions. We have thoroughly considered all the suggestions and have made suitable corrections in the manuscript. The changes in the revised manuscript are indicated in red font. Responses pertaining to the reviewers' comments are given below:

Reviewer #4 (Remarks to the Author):

Comment : *I'm happy with the changes, but the new paragraph 317-325 needs to be proofread, e.g. :*

317 The difference in USP35 activity according E3 ligases: change 'according' to ' with respect to'

319 is important to the correct localization: change 'to' to 'for'

323 requires for removing: change 'for removing' to 'to remove'

Answer : Thanks for your comments. We changed these words following the suggestions.
